# Supplementary material for: Blood pressure control in patients aged above and below 75 years
Source: PLoS One. 2024 Feb 1;19(2):e0297103. doi: 10.1371/journal.pone.0297103 (PMC10833546; doi:10.1371/journal.pone.0297103)
Supplement: S1 Appendix — (DOCX) [file pone.0297103.s001.docx]

**S1 Appendix. Covariates**

Outlier data were excluded from the health screening records.

(a) serum creatinine <0.3 mg/dl or >15.0 mg/dl

(b) total cholesterol <130 mg/dl or >320 mg/dl

(c) HDL cholesterol <20 mg/dl or >100 mg/dl

(d) body mass index <10.0 kg/m^2^ or >50.0 kg/m^2^

(e) alcohol drinks per drinking day >35 drinks

(f) systolic blood pressure (SBP) <90 mm Hg or >200 mm Hg

(g) fasting blood glucose <30 mg/dl or >900 mg/dl

**A. Fixed covariates**

Nationwide health screenings were performed generally at 2-year intervals. The baseline variables of total cholesterol, body mass index, income level, and drinking amount were determined as averaged values for 2005–2010. The variables of serum creatinine, HDL cholesterol, and waist circumference were determined using the values measured in 2009 or 2010. Albuminuria was determined with urine dipstick results between 2007 and 2010.

The eGFR was calculated from serum creatinine using the Chronic Kidney Disease Epidemiology Collaboration equation [1] and was categorized into 6 levels (15.0–29.9, 30.0–44.9, 45.0–59.9, 60.0–89.9, 90.0–119.9, or ≥120 ml/min/1.73 m^2^). Dipstick albuminuria was categorized into 3 levels (negative, 1+ at least once or trace twice, or ≥2+ at least once).

End-stage kidney disease was determined as dialysis for ≥90 days per year or kidney transplantation (S1 Table).

Total cholesterol was categorized into 5 levels (130–159, 160–199, 200–239, 240–279, or 280–320 mg/dl), and HDL cholesterol into 3 levels (20.0–39.9, 40.0–59.9, or 60.0–100.0 mg/dl), based on the ATP III classifications [2].

Body mass index was categorized into 5 levels (10.0–18.4, 18.5–22.9, 23.0–24.9, 25.0–29.9, or 30.0–49.9 kg/m^2^) referring to the WHO classifications [3]. Waist circumference was categorized into 3 levels (<85.0, 85.0–89.9, or ≥90.0 cm in men; <80.0, 80.0–84.9, or ≥85.0 cm in women) referring to the publication for Asian populations [4–6].

Income levels were determined by income-based insurance contributions. The vigintile values of insurance contributions were averaged from 2005 to 2010. The averaged values were categorized into 5 levels (1–4, 5–8, 9–12, 13–16, or 17–20).

Smoking status was categorized into 3 groups (never, former, and current smoker). Exercise frequency was categorized into 4 levels (<1, 1–2, 3–4, or ≥5 days per week). Drinking amount was categorized into 6 levels (0.0, 0.1–3.4, 3.5–7.4, 7.5–14.4, 14.5–28.4, or ≥28.5 drinks per week) with log-scale intervals incorporating the cutoff values for the NIAAA’s definition of low-risk drinking [7].

Medical history of cardiovascular disease was determined by NHIS reimbursement records of revascularization or critical care unit admission (S1 Table) for acute coronary syndrome (I21–I24) or acute ischemic stroke (I63–I66) or a health screening questionnaire for history of heart disease or stroke.

Hypoglycemic and statin treatment statuses were determined using NHIS reimbursement records for the prescription of drugs (S1 Table) and categorized into 3 groups (no or occasional, irregular, or regular medication). Regular medication was considered if the prescription was for >2/3 of the period from the initiation of medication to the baseline, irregular medication if the prescription was for 1/3 to 2/3 of each period, no or occasional medication if the prescription was for <1/3 of each period.

Fasting blood glucose was classified into 8 categories (30–79, 80–99, 100–109, 110–125, 126–139, 140–159, 160–179, or 180–900 mg/dl) incorporating cutoffs for prediabetes and diabetes [8].

**B. Risk factors**

Participants were classified into 3 risk categories by the number of additional risk factors (≥2, 1, or 0) present at baseline. Using baseline data, 4 additional risk factors (diabetes, dyslipidemia, albuminuria, and active smoking) were identified: (i) diabetes as fasting blood glucose ≥126 mg/dl or a prescription of hypoglycemic agents for ≥90 days per year (S1 Table). (ii) dyslipidemia as total cholesterol ≥240 mg/dL, HDL cholesterol <40 mg/dL, or prescription of statins for ≥90 days per year. (iii) albuminuria as urine dipstick albumin ≥1+ at least once or ≥trace twice. (iv) active smoking as current smoking.

**C. Time-varying covariates**

In each year of follow-up, antihypertensive treatment status was determined and categorized into 3 groups (occasional, irregular, or regular medication). Regular medication was considered if the prescription was for >2/3 of the period from the initiation of medication to each year, irregular medication if the prescription was for 1/3 to 2/3 of each period, no or occasional medication if the prescription was for <1/3 of each period.

On-treatment and untreated BP values were separated as the risk threshold was quite different depending on treatment status [9,10]. The on-treatment BP was considered as the case that received antihypertensive agents for ≥90 days in the year of measurement. In each year, SBP values were averaged from the cohort entry (year 2005). The timely-averaged SBP was classified into 8 categories (90–104, 105–114, 115–124, 125–134, 135–144, 145–154, 155–164, or 165–200 mm Hg).

**References**

1. Levey AS, Stevens LA, Schmid CH, Zhang YL, Castro AF 3rd, Feldman HI, et al. A new equation to estimate glomerular filtration rate. Ann Intern Med. 2009;150: 604–612.

2. Expert Panel on Detection E and Treatment of High Blood Cholesterol in Adults. Executive Summary of The Third Report of The National Cholesterol Education Program (NCEP) Expert Panel on Detection, Evaluation, And Treatment of High Blood Cholesterol In Adults (Adult Treatment Panel III). JAMA. 2001;285: 2486–2497.

3. WHO Expert Consultation. Appropriate body-mass index for Asian populations and its implications for policy and intervention strategies. Lancet. 2004;363: 157–163. doi:10.1016/S0140-6736(03)15268-3

4. Bao Y, Lu J, Wang C, Yang M, Li H, Zhang X, et al. Optimal waist circumference cutoffs for abdominal obesity in Chinese. Atherosclerosis. 2008;201: 378–384. doi:10.1016/j.atherosclerosis.2008.03.001

5. Lear SA, James PT, Ko GT, Kumanyika S. Appropriateness of waist circumference and waist-to-hip ratio cutoffs for different ethnic groups. Eur J Clin Nutr. 2010;64: 42–61. doi:10.1038/ejcn.2009.70

6. Ahmad N, Adam SIM, Nawi AM, Hassan MR, Ghazi HF. Abdominal Obesity Indicators: Waist Circumference or Waist-to-hip Ratio in Malaysian Adults Population. Int J Prev Med. 2016;7: 82. doi:10.4103/2008-7802.183654

7. Alcohol Facts and Statistics. In: National Institute on Alcohol Abuse and Alcoholism (NIAAA) [Internet]. 25 Apr 2019 [cited 9 Nov 2019]. Available: https://www.niaaa.nih.gov/publications/brochures-and-fact-sheets/alcohol-facts-and-statistics

8. Organization WH, Others. International Diabetes Federation (2006) Definition and diagnosis of diabetes mellitus and intermediate hyperglycemia: report of a WHO/IDF consultation. IDF Consult. 2008.

9. Jung HH, Park JI, Jeong JS. Blood Pressure-Related Risk Among Users Versus Nonusers of Antihypertensives: A Population-Based Cohort in Korea. Hypertension. 2018;71: 1047–1055. doi:10.1161/HYPERTENSIONAHA.118.11068

10. Jung HH. Association of Optimal Blood Pressure With Critical Cardiorenal Events and Mortality in High-Risk and Low-Risk Patients Treated With Antihypertension Medications. JAMA Netw Open. 2019;2: e199307–e199307. doi:10.1001/jamanetworkopen.2019.9307
