# Supplementary material for: Blood pressure control in patients aged above and below 75 years
Source: PLoS One. 2024 Feb 1;19(2):e0297103. doi: 10.1371/journal.pone.0297103 (PMC10833546; doi:10.1371/journal.pone.0297103)
Supplement: S1 Table — (DOCX) [file pone.0297103.s002.docx]

**S1 Table. NHIS codes for patient management and diagnosis**

|  | **NHIS Code** |
| --- | --- |
| **Antihypertensive agents^*^** | ACE inhibitor, 1042, 1147, 1229, 1330, 1409, 1516, 1635, 1734, 1845, 1968, 2113, 2219, 2224, 2310, 2350, 2351, 2424, 2622, 2623, 3787, 3859, 4028, 4403, 4407, 4408, 4470, 4471, 4472, 4486, 4487, 4536, 4537, 4660, 4901, 4979, 4992, 4993, 5016, 5104, 5562;  AG II antagonist, 1226, 1773, 1857, 2471, 2625, 3564, 3788, 3789, 3857, 3858, 4237, 4292, 4426, 4432, 4433, 4605, 4685, 4774, 4869, 4891, 4928, 4929, 4958, 5005, 5006, 5026, 5027, 5030, 5115, 5116, 5117, 5136, 5139, 5152, 5197, 5198, 5199, 5200, 5201, 5209, 5212, 5213, 5214, 5220, 5222, 5223, 5224, 5226, 5227, 5228, 5229, 5230, 5231, 5232, 5233, 5234, 5240, 5241, 5250, 5251, 5252, 5253, 5263, 5264, 5265, 5268, 5269, 5270, 5271, 5475, 5476, 5477, 5478, 5479, 5480, 5822, 5824, 6231, 6294, 6295, 6296, 6297, 6298, 6299, 6300, 6301, 6302, 6313, 6316, 6317, 6328, 6329, 6330, 6349, 6350, 6351, 6352, 6374, 6375, 6376, 6441, 6442, 6448, 6519, 6520, 6521, 6527, 6529, 6530, 6531, 6624, 6628, 6629, 6630, 6635, 6636, 6637, 6638, 6735, 6736, 6827, 6828, 6829;  Ca-antagonist, 1076, 1140, 1151, 1331, 1457, 1575, 1789, 1803, 1820, 1880, 2010, 2011, 2017, 2024, 2476, 2624, 3562, 4412, 4470, 4471, 4472, 4598, 4599, 4646, 4660, 4708, 4723, 4724, 4725, 4762, 4797, 4832, 4865, 4928, 4929, 4958, 4959, 5005, 5006, 5027, 5030, 5115, 5116, 5117, 5139, 5189, 5197, 5198, 5199, 5200, 5201, 5212, 5213, 5214, 5222, 5223, 5224, 5226, 5227, 5228, 5229, 5230, 5231, 5232, 5233, 5234, 5282, 5475, 5476, 5477, 5478, 5479, 5480, 5822, 5824, 6145, 6231, 6294, 6295, 6296, 6313, 6328, 6329, 6330, 6374, 6375, 6376, 6448, 6519, 6520, 6521, 6527, 6529, 6530, 6531, 6628, 6629, 6630, 6635, 6636, 6637, 6638, 6827, 6828, 6829;  Central sympatholytic, 1365, 1927, 1975, 2231, 2631;  Direct vasodilator, 1707, 1961, 2293, 4236, 4606;  Thiazide, 1708, 1744, 2447, 2620, 2621, 2622, 2623, 2625, 2626, 2627, 2628, 2630, 3564, 3787, 3789, 3857, 3858, 4237, 4273, 4274, 4403, 4407, 4408, 4426, 4432, 4433, 4486, 4487, 4513, 4536, 4537, 4554, 4555, 4605, 4698, 4699, 4700, 4774, 4869, 4891, 4901, 4979, 4992, 4993, 5026, 5136, 5197, 5198, 5200, 5220, 5268, 5562, 6628, 6629, 6630, 6635, 6636, 6637, 6638, 6735, 6736, 6827, 6828, 6829;  α antagonist, 1200, 1491, 1749, 2168, 2355, 2629, 4414, 4834;  β blocker, 1079, 1114, 1168, 1170, 1179, 1250, 1370, 1802, 2023, 2098, 2620, 2621, 2624, 2626, 4273, 4274, 4554, 4555, 4601, 4602, 4698, 4699, 4700, 4831, 4895 |
| **Hypoglycemic agents^*^** | DPP-4 inhibitor, 5008, 5011, 5023, 5029, 5070, 5071, 5137, 5185, 5186, 5196, 5205, 5206, 5207, 5238, 5247, 6133, 6164, 6191, 6242, 6273, 6303, 6304, 6305, 6306, 6320, 6356, 6357, 6396, 6418, 6419, 6420, 6450, 6453, 6484, 6485, 6486, 6499, 6500, 6501, 6541, 6646, 6647, 6648;  Glitazone, 3480, 4319, 4527, 4529, 4612, 4691, 4718, 4888, 4889, 4890, 4981, 5255, 5256, 5259, 6303, 6304, 6305, 6306, 6538, 6539, 6540, 6541, 6557;  GLP-1 agonist, 5121, 6266, 6397, 6398, 6414, 6445, 6667, 6670  Insulin, 1183, 1701, 1702, 1703, 1704, 1705, 1706, 1752, 1753, 2156, 2157, 3278, 4413, 4618, 4849, 4887, 5074, 6267, 6268, 6667, 6670;  Meglitinide, 3795, 4302, 4861, 5188, 6319, 6321, 6372, 6449;  Metformin, 1915, 4211, 4434, 4435, 4527, 4529, 4612, 4691, 4718, 4719, 4742, 4743, 4972, 4981, 4986, 5023, 5029, 5070, 5071, 5137, 5185, 5186, 5188, 5196, 5205, 5206, 5207, 5236, 5237, 5238, 5247, 6319, 6320, 6321, 6356, 6357, 6372, 6398, 6414, 6418, 6419, 6420, 6449, 6450, 6484, 6485, 6486, 6490, 6491, 6492, 6493, 6494, 6495, 6499, 6500, 6501, 6538, 6539, 6540, 6541, 6557, 6718, 6719, 6720, 6721, 6725, 6726, 6727, 6728, 6729, 6730, 6738, 6833, 6834;  SGLT2 inhibitor, 5273, 6282, 6361, 6398, 6414, 6490, 6491, 6492, 6493, 6494, 6495, 6743;  Sulfonylurea, 1320, 1654, 1655, 1656, 1657, 1658, 1659, 4174, 4211, 4434, 4435, 4719, 4742, 4743, 4888, 4889, 4890, 4972, 4986, 5255, 5256;  α-glucosidase inhibitor, 1006, 2490, 4062, 5236, 5237 |
| **Statin^*^** | Atorvastatin, 1115, 4723, 4724, 4725, 5022, 5189, 5240, 5241, 5270, 5271, 6145, 6338, 6339, 6346, 6348, 6718, 6719, 6720, 6721, 6738;  Cerivastatin, 1304;  Fluvastatin, 1624;  Lovastatin, 1858, 1859;  Pitavastatin, 4709, 6349, 6350, 6351, 6352, 6793;  Pravastatin, 2166, 5193;  Rosuvastatin, 4540, 5250, 5251, 5252, 5253, 5263, 5264, 5265, 5269, 6297, 6298, 6299, 6300, 6301, 6302, 6316, 6317, 6407, 6408, 6409, 6441, 6442, 6634, 6646, 6647, 6648, 6725, 6726, 6727, 6728, 6729, 6730, 6833, 6834;  Simvastatin, 2278, 4710, 4711, 5078, 6314, 6315 |
| **Cholinesterase inhibitors and memantine** | Donepezil, 1486, 6434; Rivastigmine, 2245; Galantamine, 3852; Memantine, 1900 |
| **CCU admission** | NHIS clause code, 02; and NHIS item code, 03 |
| **Coronary revascularization** | Percutaneous coronary angioplasty, M6551, M6552, M6553, M6554;  Percutaneous coronary stent insertion, M6561, M6562, M6563, M6564, M6565, M6566, M6567;  Percutaneous coronary thrombolysis or thrombectomy, M6634, M6638;  Percutaneous coronary atherectomy, M6571, M6572;  Coronary artery bypass graft, O1647, OA641, OA642;  Coronary artery endarterectomy, O1830 |
| **Cerebral or carotid revascularization** | Percutaneous cerebral angioplasty, M6593, M6599;  Percutaneous cerebral stent insertion, M6601;  Percutaneous cerebral thrombolysis or thrombectomy, M6630, M6636;  Percutaneous carotid angioplasty, M6594;  Percutaneous carotid stent insertion, M6602;  Percutaneous carotid thrombolysis or thrombectomy, M6635, M6637;  Transluminal carotid atherectomy, O0226;  Carotid endarterectomy, O0227 |
| **Renal replacement therapy** | Haemodialysis, O7020, O9991;  Peritoneal dialysate, 3214, 3216, 3218, 3220, 3221, 3222, 3223, 3224, 3225, 3226, 3240, 3243, 3244, 3245, 3248, 3249, 3250, 3251, 3252, 3253, 3254, 3255, 3256, 3257, 3259, 3260, 3496, 3497, 3498, 3499, 3500, 3501, 3502, 3503, 3506, 3507, 3508, 3509, 3510, 3511, 3512, 3513, 3514, 3515, 3516, 3517, 3518, 3519, 3520, 3521, 3522, 3523, 3524, 3525, 3526, 3527, 3528, 3529, 3530, 3531, 3532, 3533, 3534, 3535, 3536, 3537, 3538, 3539, 3540, 3541, 3601, 3602, 3603, 3659, 3660, 3661, 3662, 3663, 3666, 3667, 3668, 3669, 3808, 3809, 4008, 4009, 4010, 4095, 4096, 4097, 4098, 4099, 4229, 4230, 4231, 4232, 4234, 4235, 4311, 4312, 4313, 4490, 4491, 4492, 4498, 4499, 4500, 4637, 4639, 4640, 4647, 4648, 4845, 4846, 4847, 4935, 4936, 4949, 4950, 4951, 5096, 5097, 5098, 5107, 5108, 5109, 5110, 5111, 5135, 5161, 5162, 5163, 6657, 6658, 6659, 6816, 6817, 6818, 6819, 6820, 6821;  Kidney transplantation, R3280 |
| **Diagnosis** | Major cancers: Lung C34; Liver C22; Colon C18–C21; Stomach C16; Pancreas C25; Gallbladder and bile duct C23, C24; Breast C50;  Chronic kidney disease, N18; Kidney transplant status, Z94.0;  Acute coronary syndrome, I21–I24; Acute ischemic stroke, I63–I66;  Dementia, F00–F03 |

^*^ The codes for combination drugs are repeated in corresponding cells.

ACE, angiotensin-converting-enzyme; AG, angiotensin; CCU, critical care unit; DPP-4 dipeptidyl peptidase-4; GLP-1, glucagon-like peptide-1; NHIS, National Health Insurance Service; SGLT2, sodium-glucose transport protein 2.
