# Supplementary material for: Blood pressure control in patients aged above and below 75 years
Source: PLoS One. 2024 Feb 1;19(2):e0297103. doi: 10.1371/journal.pone.0297103 (PMC10833546; doi:10.1371/journal.pone.0297103)
Supplement: S2 Table — (DOCX) [file pone.0297103.s003.docx]

**S2 Table. Time-lagged covariates for subsequent years**

| **Year of Outcome Variable** | **Year or Period for Time-Varying Covariate** | | **Period for Fixed Covariate** |
| --- | --- | --- | --- |
| **Clinical Endpoint** | **Antihypertensive Treatment Status** | **Time-Averaged SBP** | **Baseline Value** |
|  |  |  |  |
| 2011 | 2010 | 2005–2010 | 2005–2010 |
| 2012 | 2011 | 2005–2011 | 2005–2010 |
| 2013 | 2012 | 2005–2012 | 2005–2010 |
| 2014 | 2013 | 2005–2013 | 2005–2010 |
| 2015 | 2014 | 2005–2014 | 2005–2010 |
| 2016 | 2015 | 2005–2015 | 2005–2010 |
| 2017 | 2016 | 2005–2016 | 2005–2010 |
| 2018 | 2017 | 2005–2017 | 2005–2010 |
| 2019 | 2018 | 2005–2018 | 2005–2010 |

SBP, systolic blood pressure.
