# Supplementary material for: Blood pressure control in patients aged above and below 75 years
Source: PLoS One. 2024 Feb 1;19(2):e0297103. doi: 10.1371/journal.pone.0297103 (PMC10833546; doi:10.1371/journal.pone.0297103)
Supplement: S3 Table — (DOCX) [file pone.0297103.s004.docx]

**S3 Table. Adjusted hazard ratios* and incidence rates^†^ of cardio-kidney events across on-treatment SBP levels according to age**

| **Group and SBP** | **No. of Event / Person-Yr** | **Hazard Ratio (95% CI)** | **Incidence (95% CI), per 1000 Person-Yr** |
| --- | --- | --- | --- |
| **Age ≥85 years (n=23,807)** | | | |
| 90–104 mm Hg | 92 / 1737 | 1.25 (1.01–1.54) | 47.5 (38.4–58.8) |
| 105–114 mm Hg | 297 / 6923 | 1.06 (0.93–1.20) | 40.3 (35.6–45.7) |
| 115–124 mm Hg | 946 / 23128 | 1.04 (0.96–1.13) | 39.7 (36.6–43.0) |
| 125–134 mm Hg^‡^ | 1622 / 42633 | 1.00 | 38.1 |
| 135–144 mm Hg | 1437 / 38259 | 0.99 (0.93–1.07) | 37.9 (35.2–40.6) |
| 145–154 mm Hg | 718 / 18842 | 1.03 (0.95–1.13) | 39.3 (36.0–42.9) |
| 155–164 mm Hg | 333 / 8043 | 1.12 (0.99–1.26) | 42.6 (37.8–47.9) |
| 165–200 mm Hg | 214 / 4383 | 1.34 (1.16–1.55) | 51.2 (44.3–59.1) |
| **Age 75–84 years (n=266,169)** | | | |
| 90–104 mm Hg | 408 / 15059 | 1.30 (1.17–1.43) | 23.2 (21.0–25.6) |
| 105–114 mm Hg | 1754 / 81197 | 1.09 (1.04–1.15) | 19.5 (18.6–20.5) |
| 115–124 mm Hg | 6449 / 329828 | 1.06 (1.03–1.09) | 19.0 (18.4–19.6) |
| 125–134 mm Hg^‡^ | 11785 / 660659 | 1.00 | 17.9 |
| 135–144 mm Hg | 10240 / 559555 | 1.04 (1.02–1.07) | 18.7 (18.2–19.2) |
| 145–154 mm Hg | 4972 / 253252 | 1.12 (1.09–1.16) | 20.1 (19.5–20.8) |
| 155–164 mm Hg | 2001 / 85315 | 1.34 (1.28–1.40) | 24.0 (22.8–25.1) |
| 165–200 mm Hg | 924 / 35402 | 1.49 (1.39–1.59) | 26.6 (24.8–28.4) |
| **Age 65–74 years (n=93,672)** | | | |
| 90–104 mm Hg | 50 / 4928 | 1.33 (1.00–1.77) | 9.6 (7.2–12.8) |
| 105–114 mm Hg | 295 / 33266 | 1.08 (0.95–1.22) | 7.8 (6.9–8.8) |
| 115–124 mm Hg | 1035 / 138399 | 1.01 (0.94–1.09) | 7.3 (6.8–7.9) |
| 125–134 mm Hg^‡^ | 1987 / 279456 | 1.00 | 7.2 |
| 135–144 mm Hg | 1746 / 217203 | 1.13 (1.06–1.21) | 8.2 (7.6–8.7) |
| 145–154 mm Hg | 787 / 84561 | 1.30 (1.20–1.42) | 9.4 (8.7–10.2) |
| 155–164 mm Hg | 245 / 23873 | 1.42 (1.24–1.62) | 10.2 (8.9–11.7) |
| 165–200 mm Hg | 112 / 7996 | 1.87 (1.54–2.26) | 13.5 (11.1–16.3) |
| **Age 45–64 years (n=162,744)** | | | |
| 90–104 mm Hg | 44 / 13058 | 1.18 (0.87–1.60) | 3.2 (2.4–4.3) |
| 105–114 mm Hg | 255 / 80550 | 1.16 (1.01–1.33) | 3.1 (2.7–3.6) |
| 115–124 mm Hg | 848 / 328362 | 0.99 (0.91–1.08) | 2.7 (2.5–2.9) |
| 125–134 mm Hg^‡^ | 1442 / 543811 | 1.00 | 2.7 |
| 135–144 mm Hg | 1056 / 328143 | 1.18 (1.08–1.27) | 3.2 (2.9–3.5) |
| 145–154 mm Hg | 455 / 102493 | 1.54 (1.39–1.72) | 4.2 (3.8–4.7) |
| 155–164 mm Hg | 159 / 27350 | 1.82 (1.54–2.15) | 4.9 (4.2–5.8) |
| 165–200 mm Hg | 65 / 9829 | 2.20 (1.71–2.83) | 6.0 (4.6–7.7) |

^*^ The hazard ratios were adjusted for age, sex, onset year of hypertension, history of cardiovascular disease, antihypertensive, hypoglycemic, and statin treatment statuses, untreated SBP, fasting blood glucose, total and HDL cholesterols, estimated glomerular filtration rate, albuminuria, body mass index, waist circumference, income level, smoking status, exercise frequency, and drinking amount. The analyses were conducted after exclusion of participants who developed cardio-kidney endpoints before baseline.

**^†^** The adjusted incidence rate and 95% CI was calculated by multiplying the hazard ratio and its 95% CI by a constant to make the sum of the products of incidence rates and person-years in FBG categories equal the total number of observed events.

^‡^ The on-treatment SBP 125–134 mg/dl was set as the reference.

CI, confidence interval; SBP, systolic blood pressure.
