# Supplementary material for: Blood pressure control in patients aged above and below 75 years
Source: PLoS One. 2024 Feb 1;19(2):e0297103. doi: 10.1371/journal.pone.0297103 (PMC10833546; doi:10.1371/journal.pone.0297103)
Supplement: S13 Table — (DOCX) [file pone.0297103.s014.docx]

**S13 Table. Adjusted hazard ratios* and incidence rates^†^ of overt dementia according to risk status after exclusion of participants with prior cardiovascular events**

| **Group^‡^** | **Age ≥75 Years (n=289,976)** | | | **Age 45 to 74 Years (n=256,416)** | | |
| --- | --- | --- | --- | --- | --- | --- |
| SBP | no. of event / person-yr | hazard ratio (95% CI) | incidence per 1000 person-yr (95% CI) | no. of event / person-yr | hazard ratio (95% CI) | incidence per 1000 person-yr (95% CI) |
| **≥2 More Risk Factors** | n=58,383 |  |  | n=59,975 |  |  |
| 90–104 mm Hg | 142 / 2815 | 1.13 (0.96–1.34) | 50.7 (42.9–60.0) | 21 / 4162 | 0.90 (0.58–1.40) | 6.5 (4.2–10.0) |
| 105–114 mm Hg | 737 / 15567 | 1.06 (0.98–1.14) | 47.5 (43.9–51.3) | 175 / 27755 | 1.00 (0.85–1.17) | 7.1 (6.1–8.4) |
| 115–124 mm Hg | 2968 / 61818 | 1.08 (1.03–1.13) | 48.3 (46.2–50.5) | 653 / 108661 | 0.96 (0.88–1.06) | 6.9 (6.3–7.6) |
| 125–134 mm Hg^§^ | 5511 / 122207 | 1.00 | 44.9 | 1277 / 183822 | 1.00 | 7.2 |
| 135–144 mm Hg | 4722 / 106145 | 0.99 (0.95–1.03) | 44.5 (42.7–46.2) | 882 / 122385 | 0.91 (0.83–0.99) | 6.5 (6.0–7.1) |
| 145–154 mm Hg | 2082 / 49183 | 0.94 (0.90–0.99) | 42.4 (40.3–44.6) | 342 / 44242 | 0.92 (0.81–1.03) | 6.6 (5.8–7.4) |
| 155–164 mm Hg | 770 / 17646 | 0.97 (0.90–1.05) | 43.7 (40.5–47.2) | 127 / 13039 | 1.15 (0.96–1.39) | 8.3 (6.9–9.9) |
| 165–200 mm Hg | 322 / 7654 | 0.94 (0.84–1.05) | 42.1 (37.6–47.2) | 33 / 4758 | 0.82 (0.58–1.16) | 5.8 (4.1–8.3) |
| **1 More Risk Factor** | n=123,280 |  |  | n=107,509 |  |  |
| 90–104 mm Hg | 300 / 6579 | 1.05 (0.93–1.18) | 45.2 (40.3–50.8) | 43 / 7128 | 1.35 (0.99–1.84) | 7.5 (5.5–10.1) |
| 105–114 mm Hg | 1555 / 34466 | 1.07 (1.01–1.12) | 46.0 (43.6–48.5) | 241 / 46777 | 1.14 (0.99–1.30) | 6.3 (5.5–7.2) |
| 115–124 mm Hg | 6274 / 138616 | 1.06 (1.03–1.10) | 45.9 (44.5–47.3) | 1010 / 194081 | 1.12 (1.03–1.21) | 6.2 (5.7–6.7) |
| 125–134 mm Hg^§^ | 11991 / 276298 | 1.00 | 43.2 | 1857 / 345286 | 1.00 | 5.5 |
| 135–144 mm Hg | 9514 / 237012 | 0.92 (0.90–0.95) | 39.8 (38.7–40.9) | 1405 / 229144 | 0.98 (0.92–1.06) | 5.4 (5.1–5.8) |
| 145–154 mm Hg | 4256 / 107630 | 0.92 (0.89–0.95) | 39.6 (38.2–41.0) | 545 / 77897 | 1.02 (0.93–1.13) | 5.7 (5.1–6.2) |
| 155–164 mm Hg | 1464 / 37067 | 0.93 (0.88–0.98) | 40.0 (37.9–42.2) | 157 / 21446 | 1.06 (0.90–1.24) | 5.8 (4.9–6.9) |
| 165–200 mm Hg | 628 / 15579 | 0.94 (0.87–1.02) | 40.6 (37.5–44.1) | 51 / 7163 | 1.21 (0.91–1.60) | 6.7 (5.0–8.8) |
| **No More Risk Factor** | n=108,318 |  |  | n=88,932 |  |  |
| 90–104 mm Hg | 292 / 6081 | 1.10 (0.98–1.24) | 47.5 (42.2–53.3) | 28 / 6669 | 1.10 (0.75–1.61) | 5.4 (3.7–7.9) |
| 105–114 mm Hg | 1350 / 30536 | 1.04 (0.98–1.10) | 44.6 (42.2–47.2) | 161 / 38845 | 1.00 (0.85–1.18) | 4.9 (4.2–5.8) |
| 115–124 mm Hg | 5348 / 121419 | 1.03 (1.00–1.07) | 44.5 (43.0–45.9) | 741 / 161882 | 1.09 (0.99–1.19) | 5.3 (4.9–5.8) |
| 125–134 mm Hg^§^ | 10606 / 245111 | 1.00 | 43.1 | 1396 / 289598 | 1.00 | 4.9 |
| 135–144 mm Hg | 8617 / 209094 | 0.95 (0.92–0.98) | 40.9 (39.8–42.1) | 1040 / 190819 | 0.99 (0.92–1.08) | 4.9 (4.5–5.3) |
| 145–154 mm Hg | 3872 / 95501 | 0.95 (0.91–0.98) | 40.8 (39.4–42.4) | 389 / 64232 | 1.03 (0.92–1.15) | 5.0 (4.5–5.7) |
| 155–164 mm Hg | 1301 / 32684 | 0.93 (0.88–0.99) | 40.2 (38.0–42.6) | 98 / 16612 | 1.01 (0.82–1.24) | 5.0 (4.0–6.1) |
| 165–200 mm Hg | 552 / 13988 | 0.91 (0.83–0.99) | 39.1 (35.8–42.6) | 37 / 5937 | 1.21 (0.87–1.69) | 6.0 (4.3–8.3) |

^*^ The hazard ratios were adjusted for age, sex, onset year of hypertension, history of cardiovascular disease, antihypertensive, hypoglycemic, and statin treatment statuses, untreated SBP, fasting blood glucose, total and HDL cholesterols, estimated glomerular filtration rate, albuminuria, body mass index, waist circumference, income level, smoking status, exercise frequency, and drinking amount.

**^†^** The adjusted incidence rate and 95% CI was calculated by multiplying the hazard ratio and its 95% CI by a constant to make the sum of the products of incidence rates and person-years in FBG categories equal the total number of observed events.

^‡^ Participants were divided into 3 risk categories by the number of the risk factors present at baseline: i.e., ≥2, 1, or 0 of the 4 risk factors (diabetes, dyslipidemia, albuminuria, and smoking).

^§^ The on-treatment SBP 125–134 mg/dl was set as the reference.

CI, confidence interval; SBP, systolic blood pressure.
