# Supplementary material for: Blood pressure control in patients aged above and below 75 years
Source: PLoS One. 2024 Feb 1;19(2):e0297103. doi: 10.1371/journal.pone.0297103 (PMC10833546; doi:10.1371/journal.pone.0297103)
Supplement: S1 Fig — (PDF) [file pone.0297103.s016.pdf]

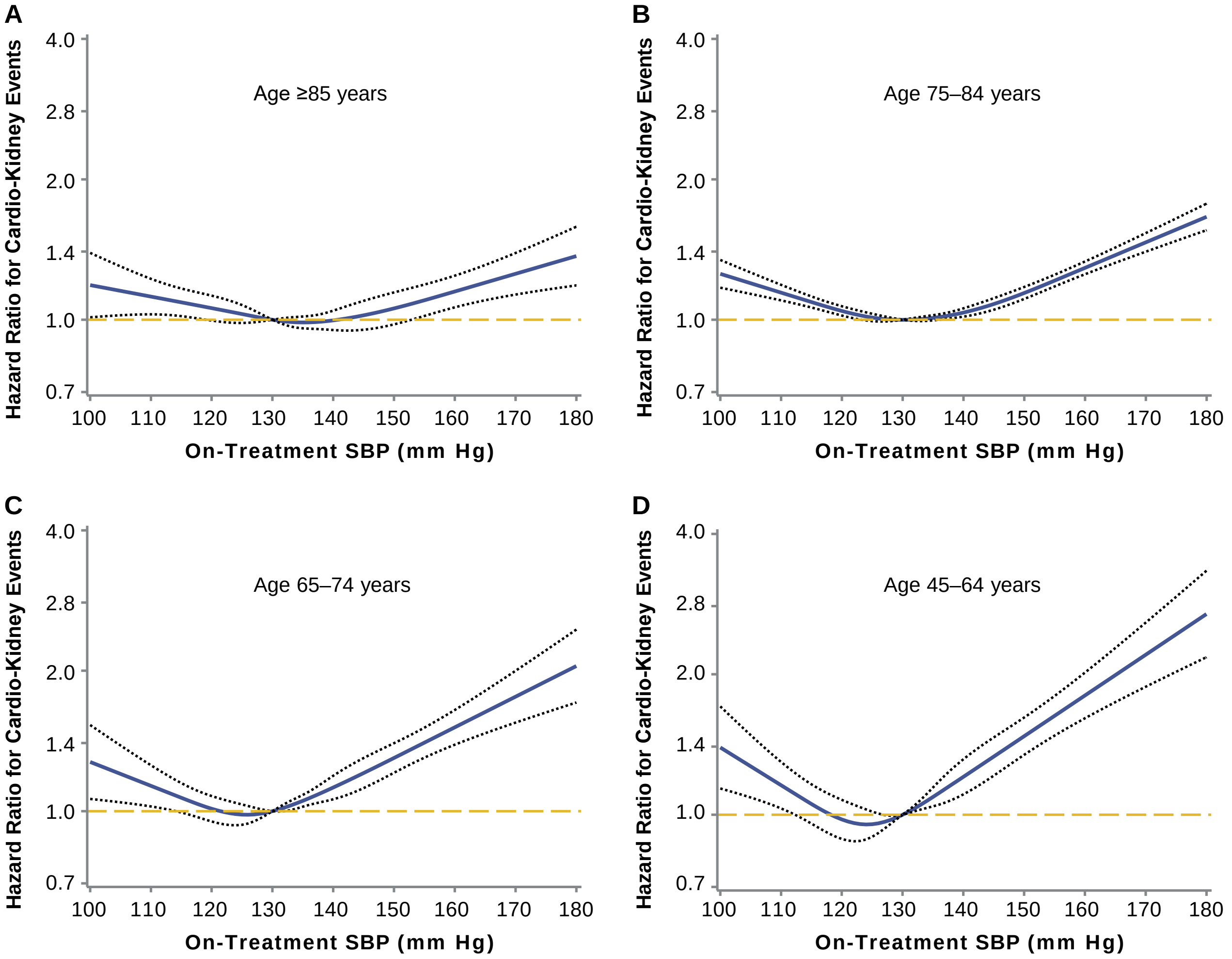
A
B
C
D
Age ≥85 years
Age 75–84 years
Age 65–74 years
Age 45–64 years
Hazard Ratio for Cardio-Kidney Events
On-Treatment SBP (mm Hg)
4.0
2.8
2.0
1.4
1.0
0.7
100
110
120
130
140
150
160
170
180

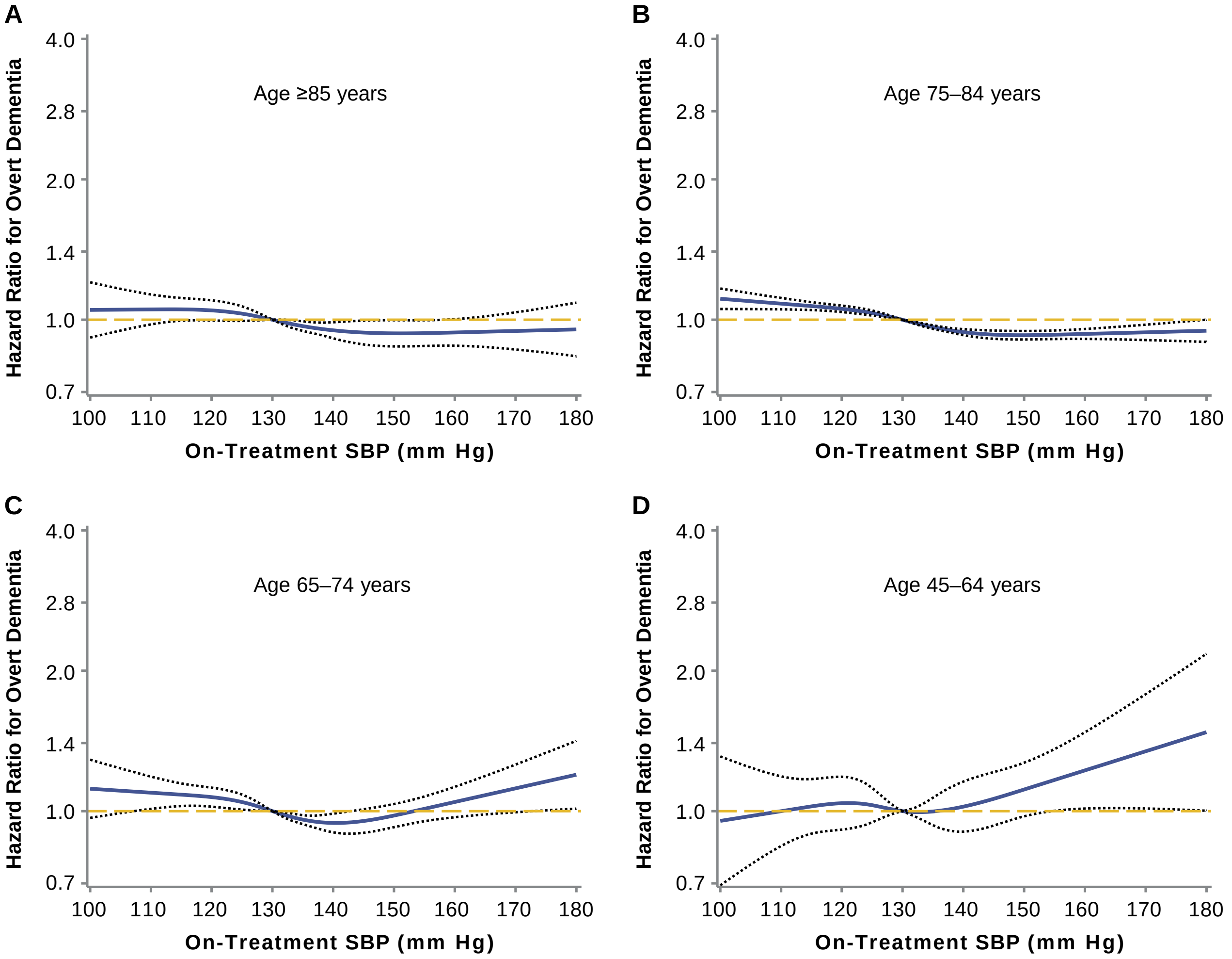
A
Age ≥85 years
Hazard Ratio for Overt Dementia
4.0
2.8
2.0
1.4
1.0
0.7
100
110
120
130
140
150
160
170
180
On-Treatment SBP (mm Hg)
B
Age 75–84 years
C
Age 65–74 years
D
Age 45–64 years

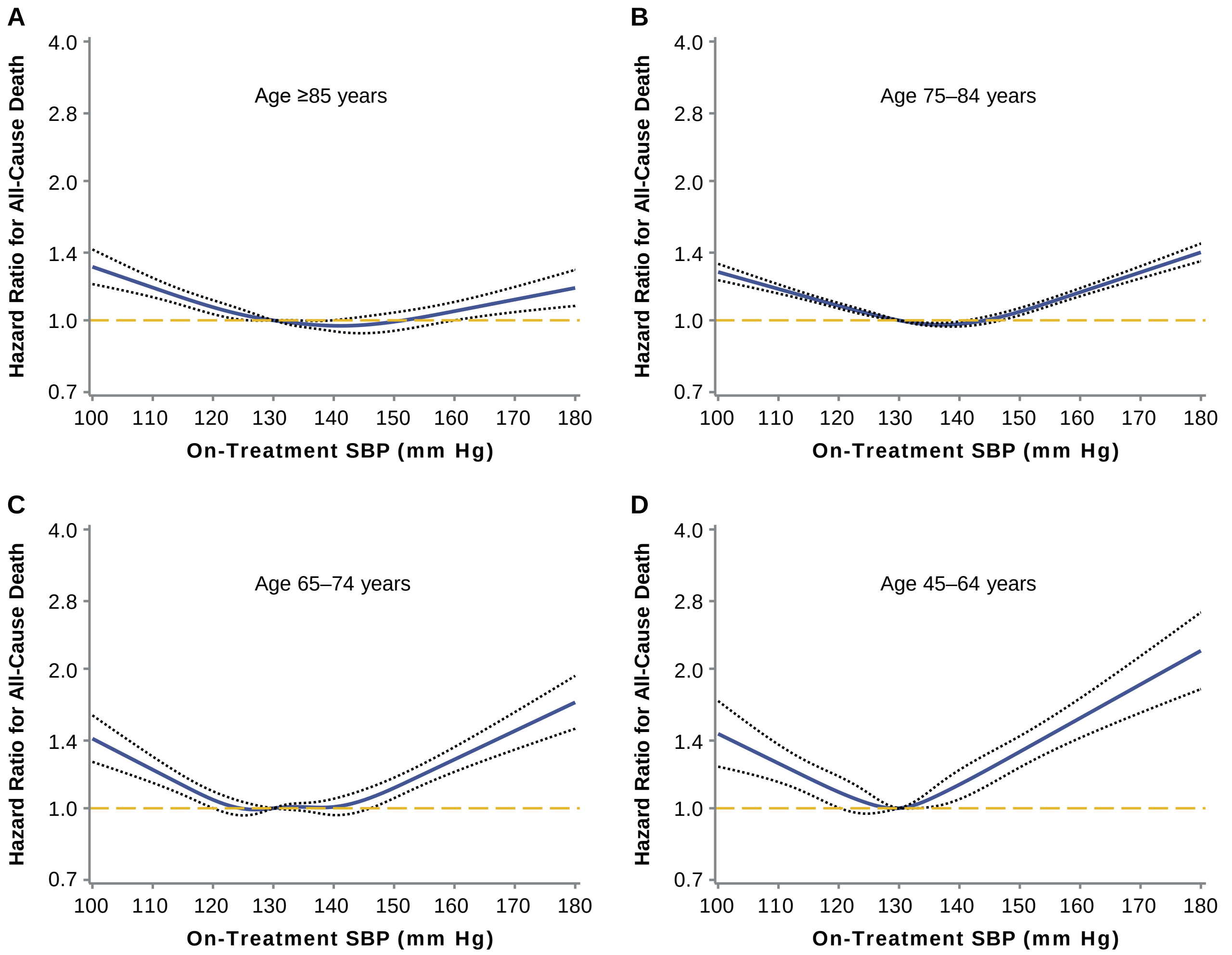

A
Age ≥85 years
B
Age 75–84 years
C
Age 65–74 years
D
Age 45–64 years
Hazard Ratio for All-Cause Death
4.0
2.8
2.0
1.4
1.0
0.7
100
110
120
130
140
150
160
170
180
On-Treatment SBP (mm Hg)

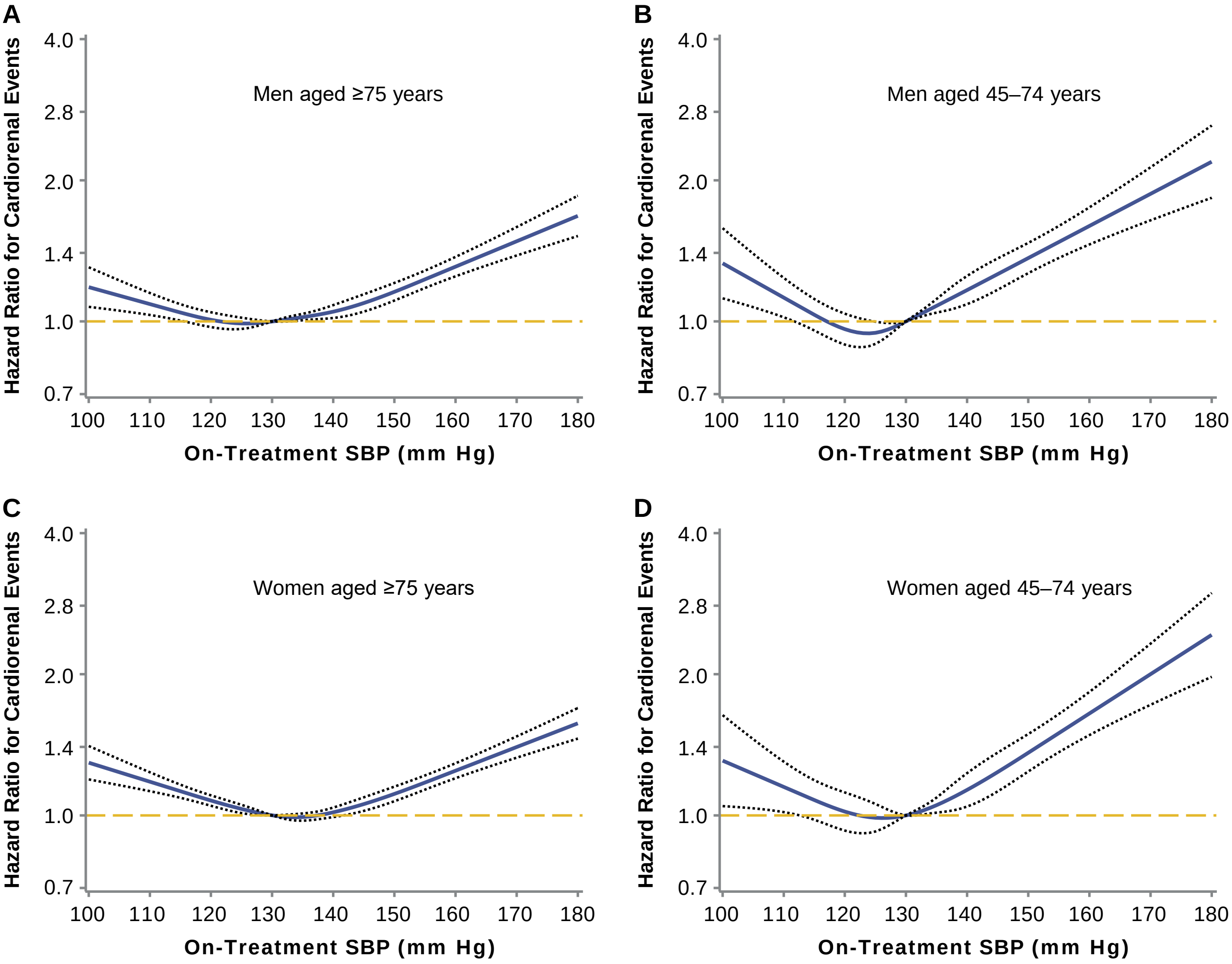
A
B
C
D
Men aged ≥75 years
Men aged 45–74 years
Women aged ≥75 years
Women aged 45–74 years
Hazard Ratio for Cardiorenal Events
On-Treatment SBP (mm Hg)
4.0
2.8
2.0
1.4
1.0
0.7
100
110
120
130
140
150
160
170
180

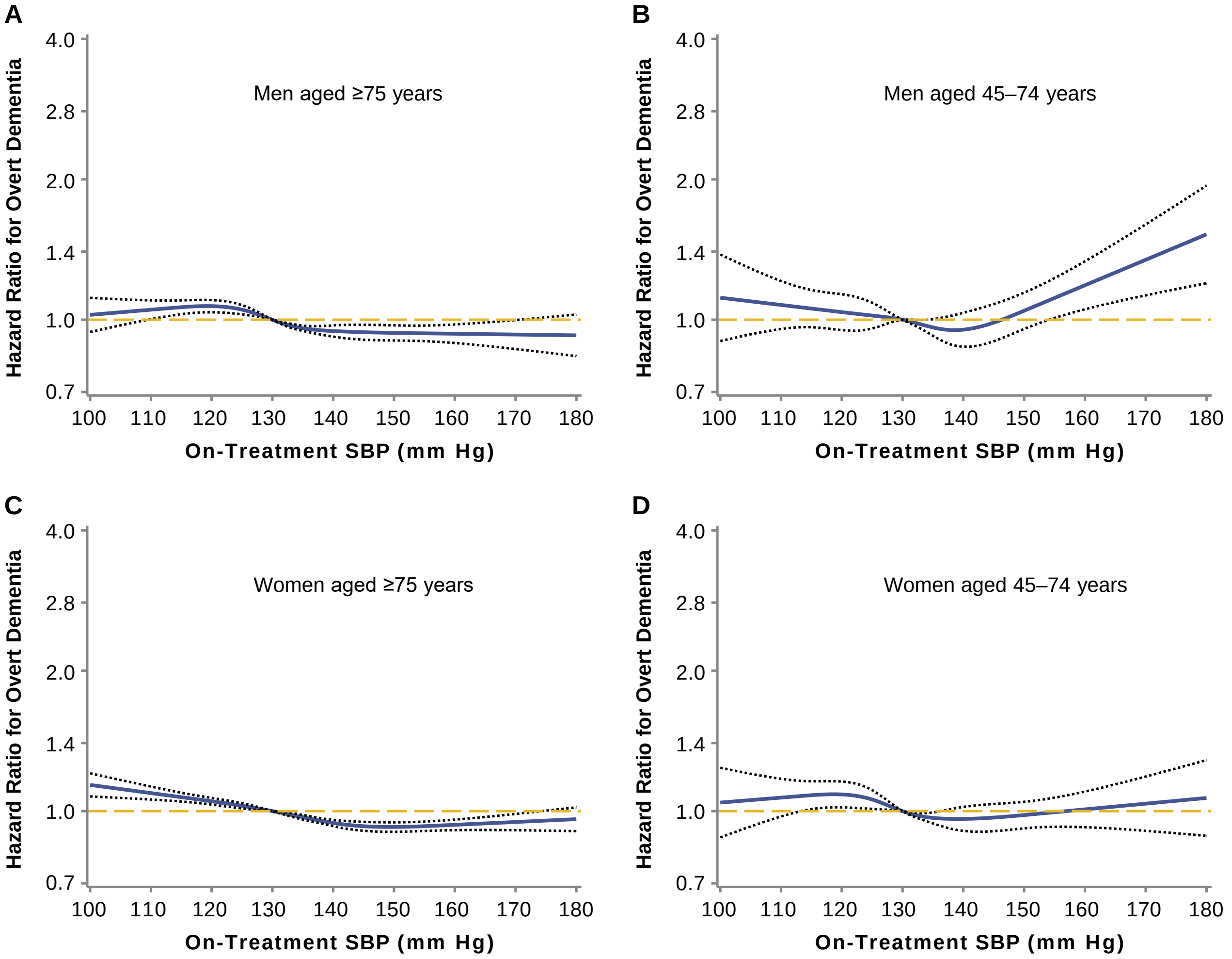
A
Men aged ≥75 years
B
Men aged 45–74 years
C
Women aged ≥75 years
D
Women aged 45–74 years
Hazard Ratio for Overt Dementia
On-Treatment SBP (mm Hg)
4.0
2.8
2.0
1.4
1.0
0.7
100
110
120
130
140
150
160
170
180

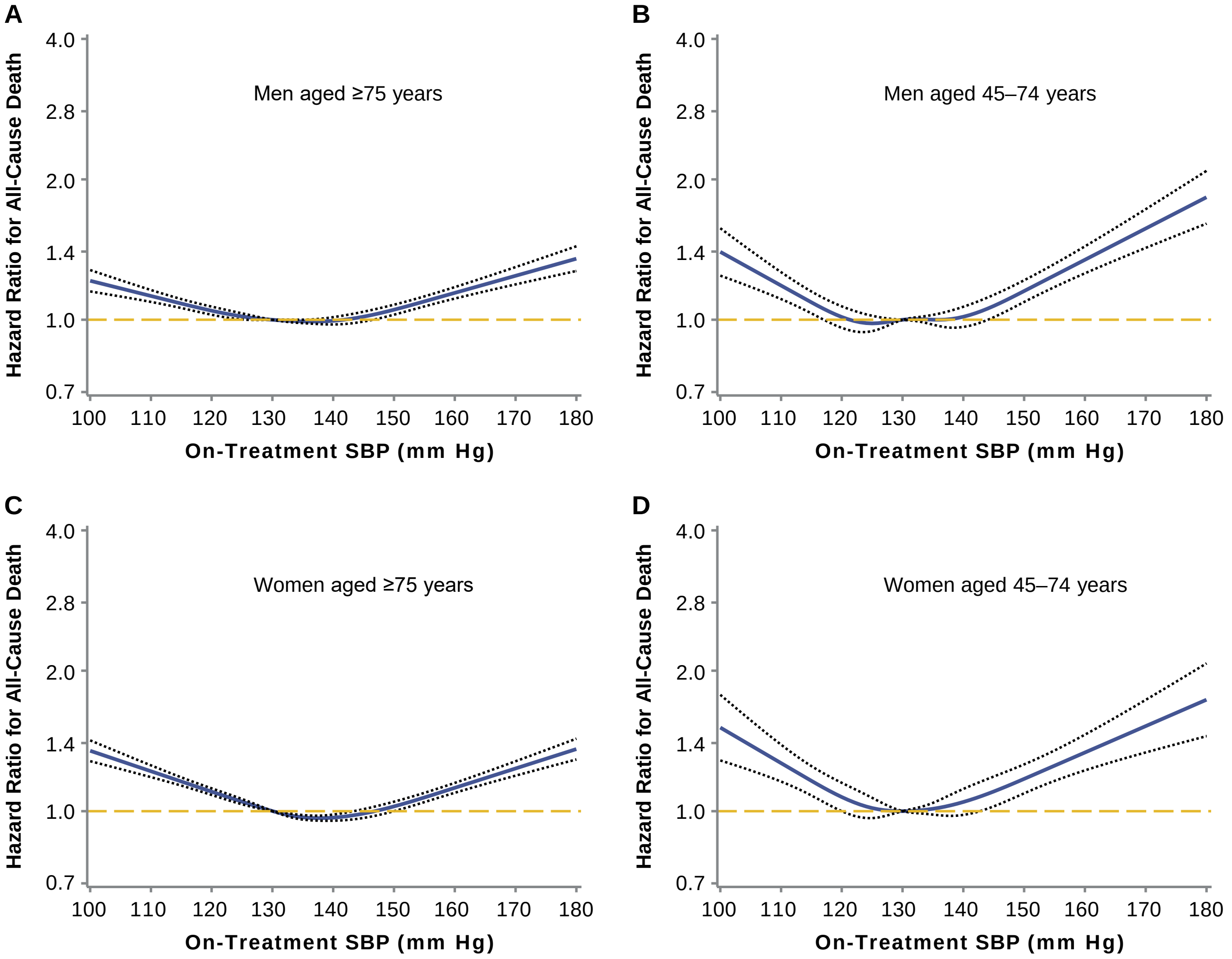
A
Men aged ≥75 years
B
Men aged 45–74 years
C
Women aged ≥75 years
D
Women aged 45–74 years
Hazard Ratio for All-Cause Death
On-Treatment SBP (mm Hg)
4.0
2.8
2.0
1.4
1.0
0.7
100
110
120
130
140
150
160
170
180
