# Supplementary figures and images for: Blood pressure control in patients aged above and below 75 years
Source: PLoS One. 2024 Feb 1;19(2):e0297103. doi: 10.1371/journal.pone.0297103 (PMC10833546; doi:10.1371/journal.pone.0297103)

**A**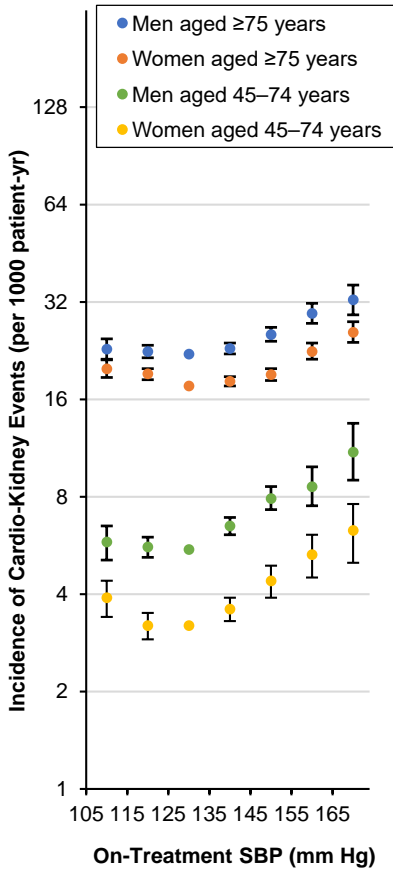**B**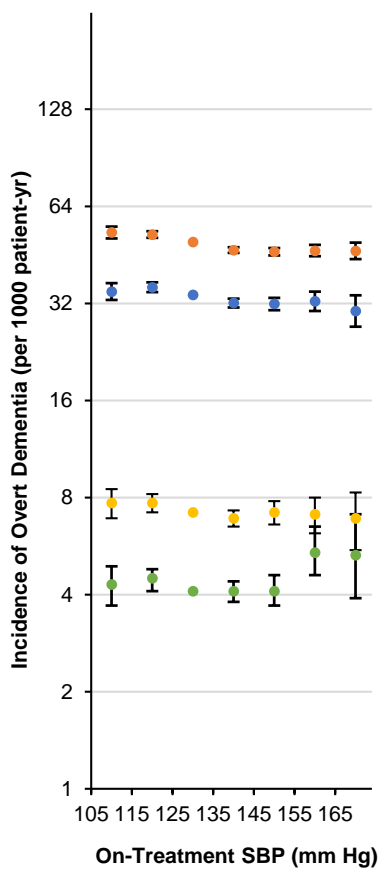**C**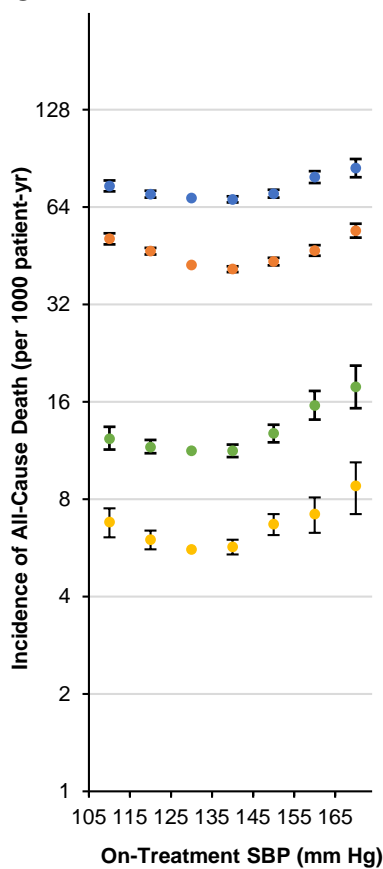

Supplement: S2 Fig — (PDF) [file pone.0297103.s017.pdf]

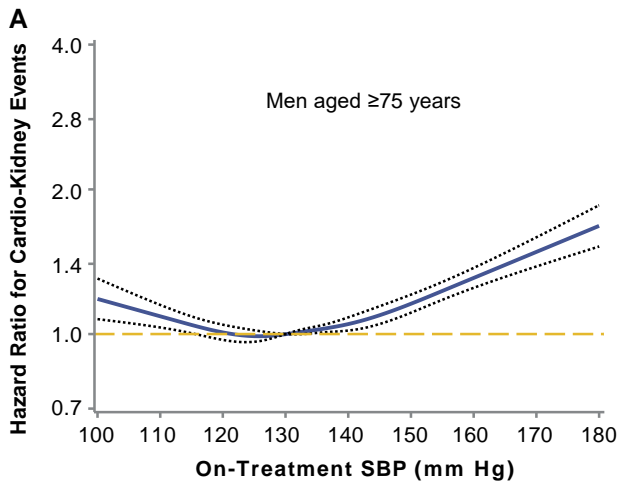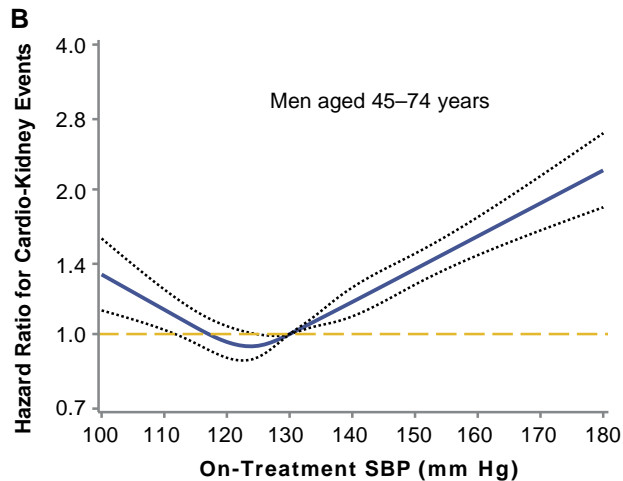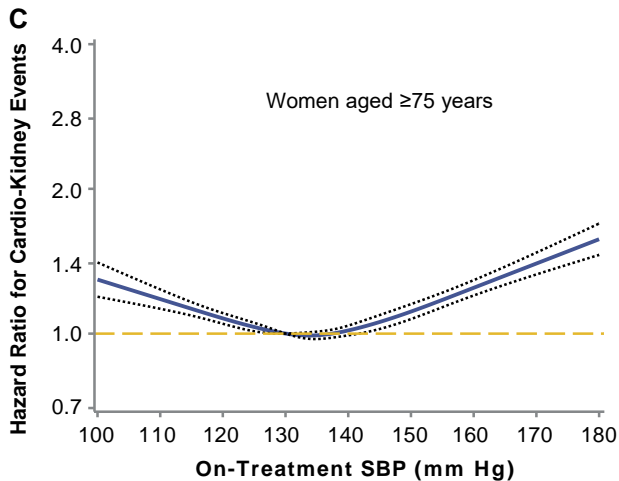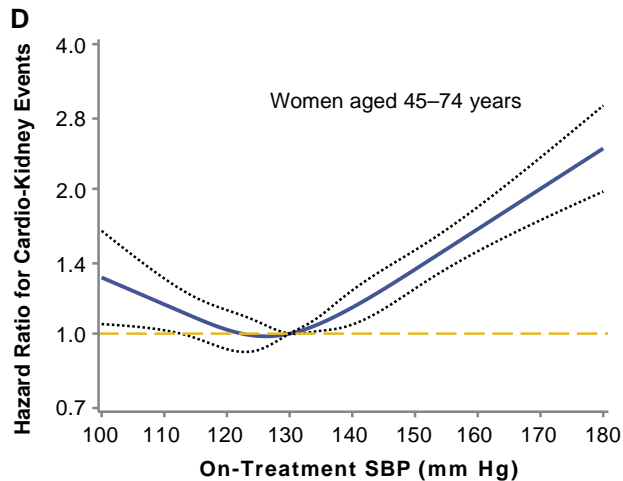

Supplement: S3 Fig — (PDF) [file pone.0297103.s018.pdf]

**A**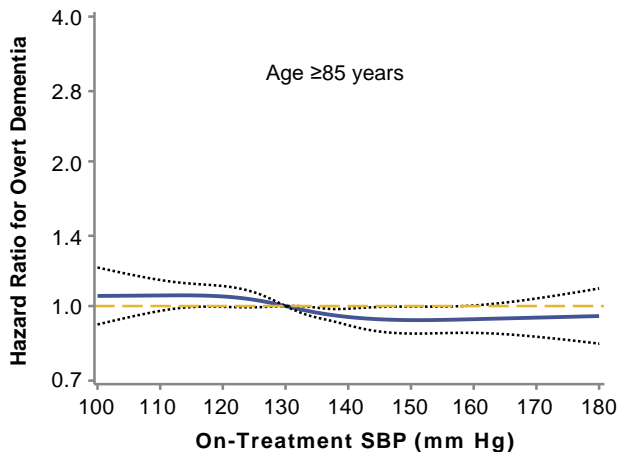**B**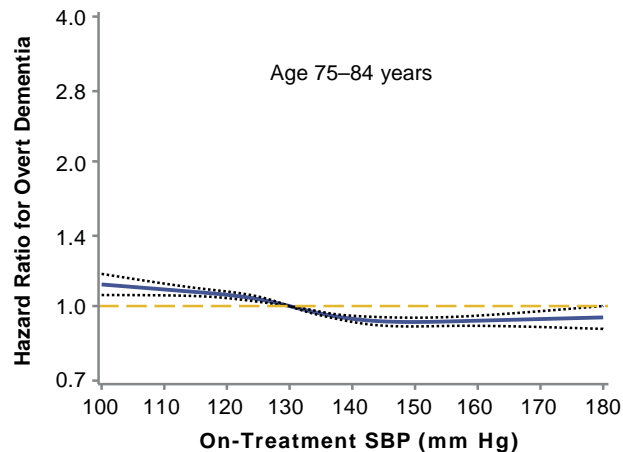**C**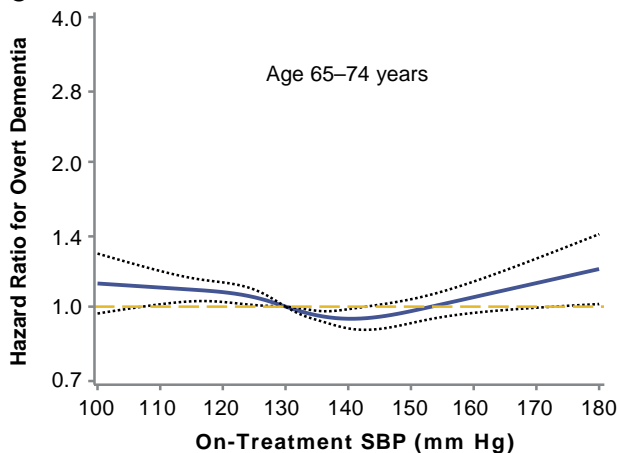**D**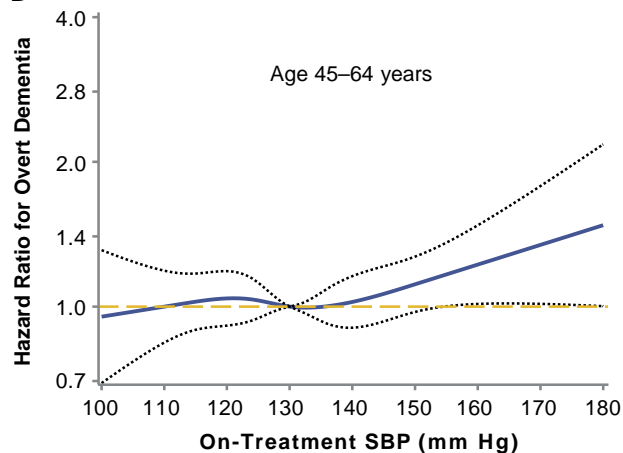

Supplement: S4 Fig — (PDF) [file pone.0297103.s019.pdf]

**A**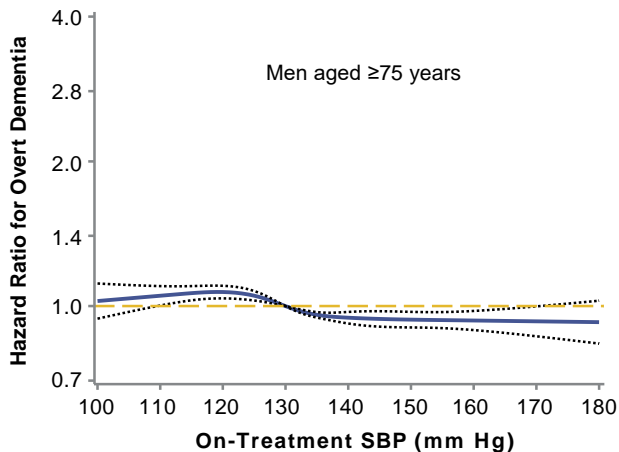**B**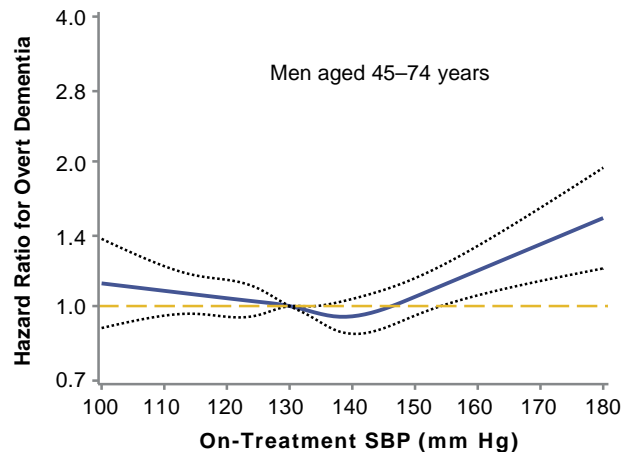**C**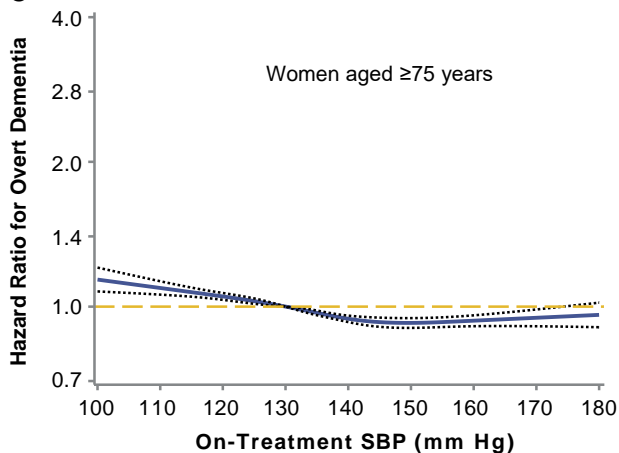**D**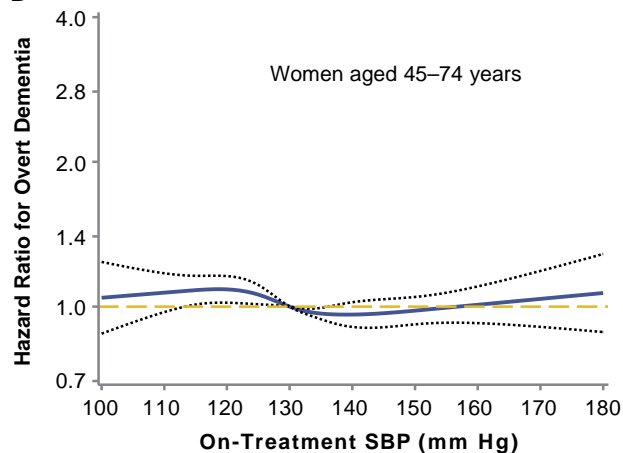

Supplement: S5 Fig — (PDF) [file pone.0297103.s020.pdf]

**A**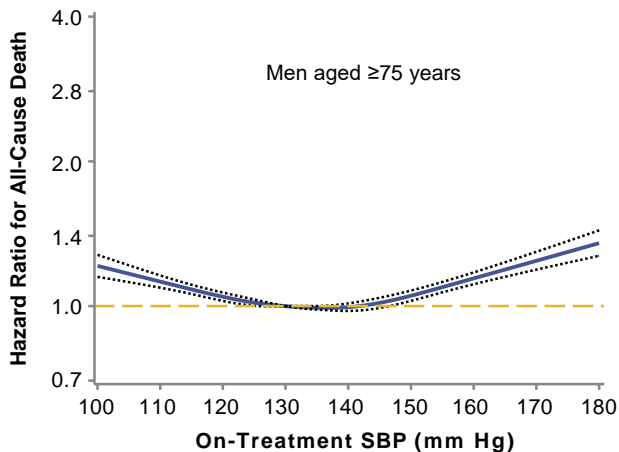**B**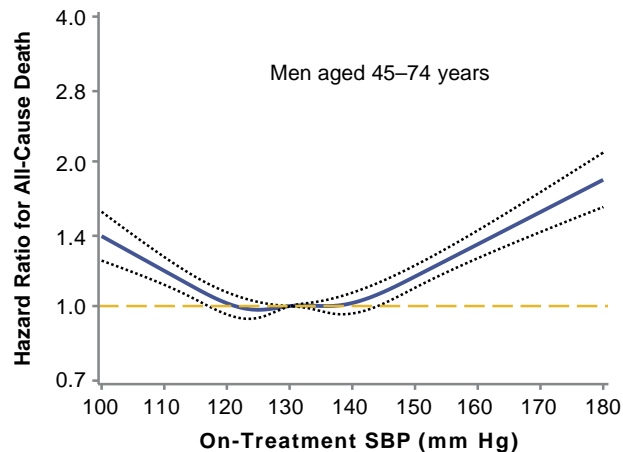**C**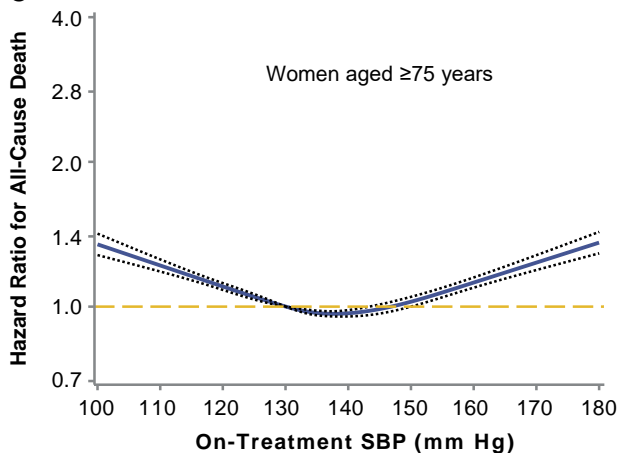**D**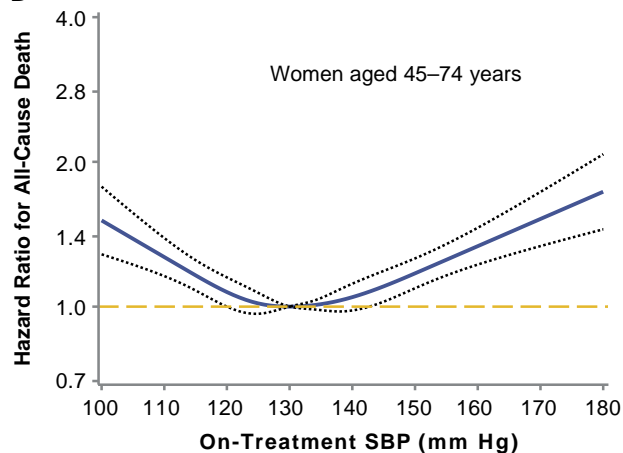

Supplement: S7 Fig — (PDF) [file pone.0297103.s022.pdf]

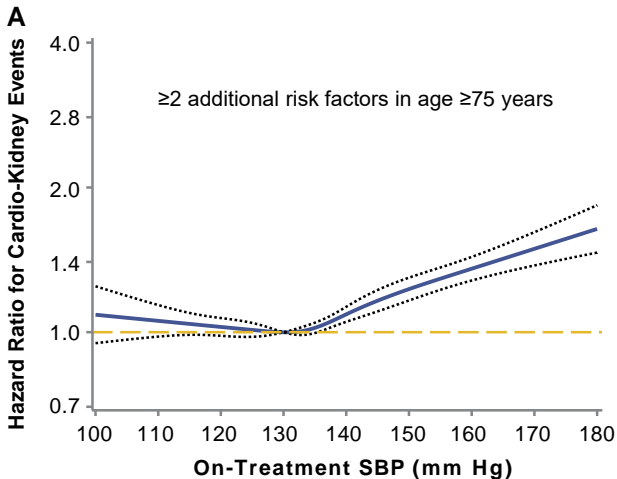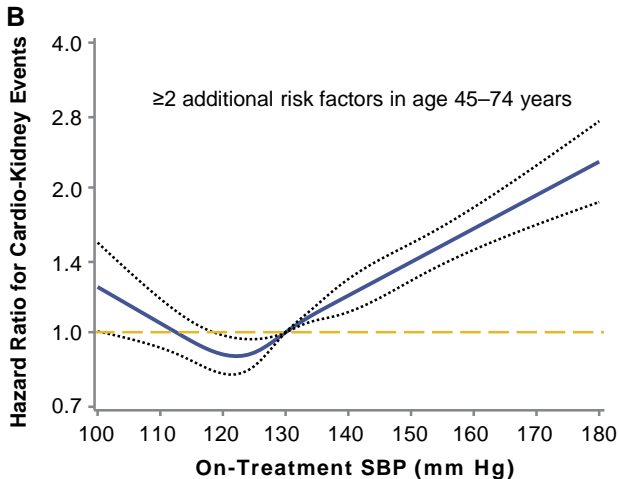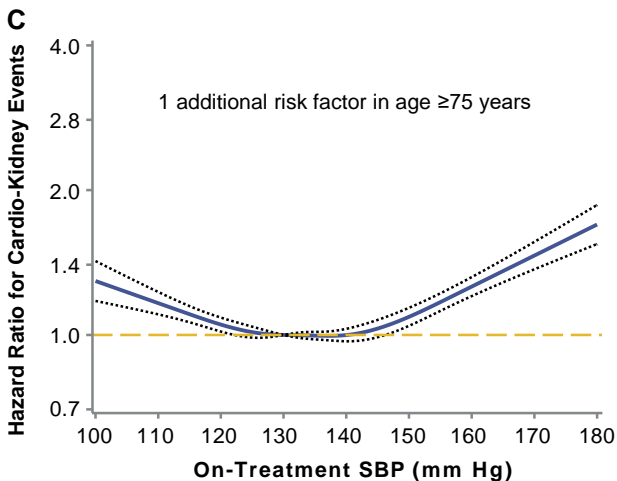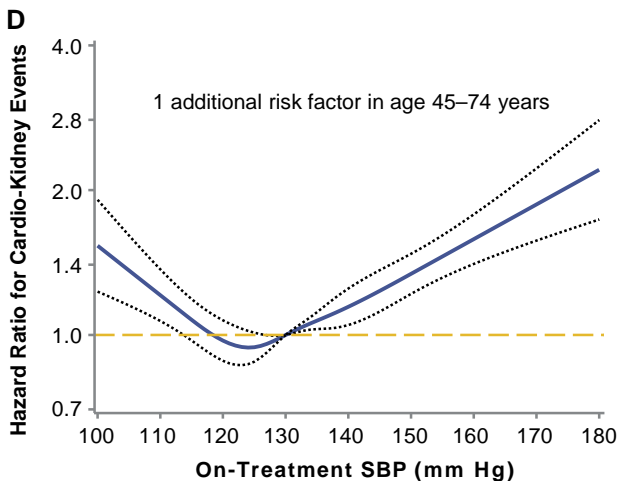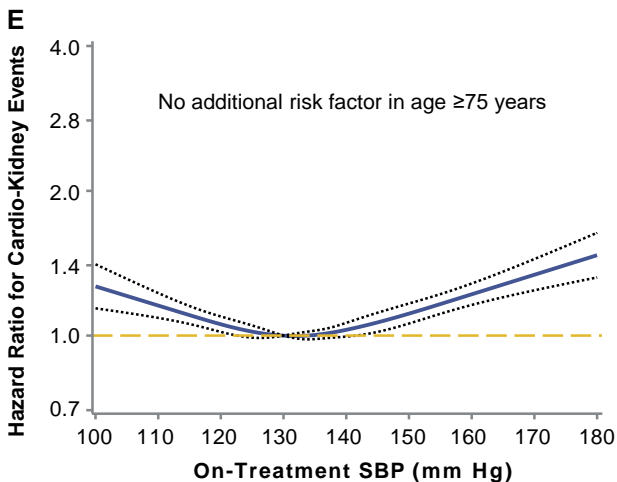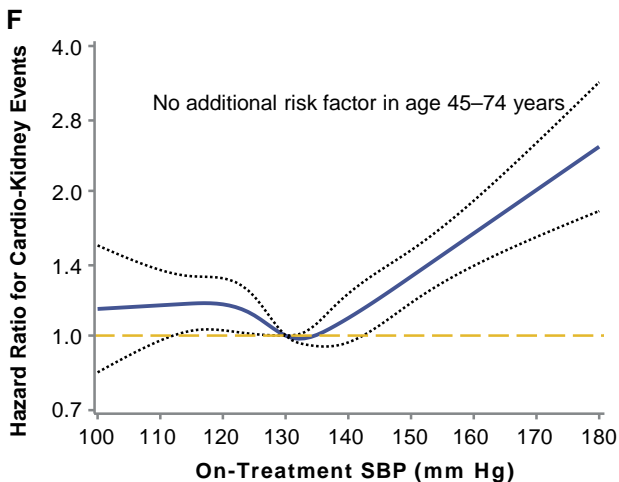

Supplement: S8 Fig — (PDF) [file pone.0297103.s023.pdf]

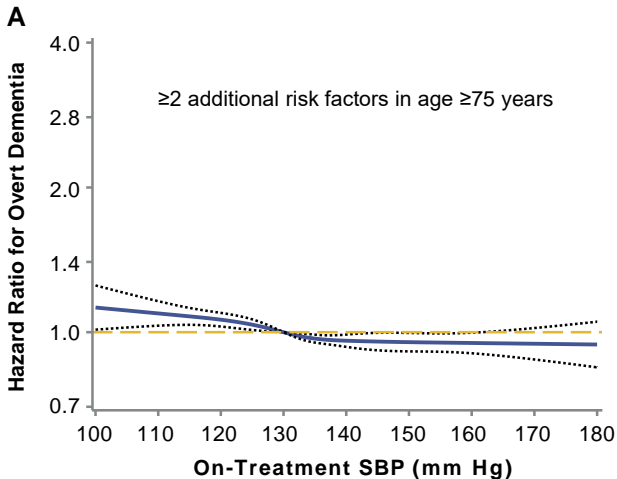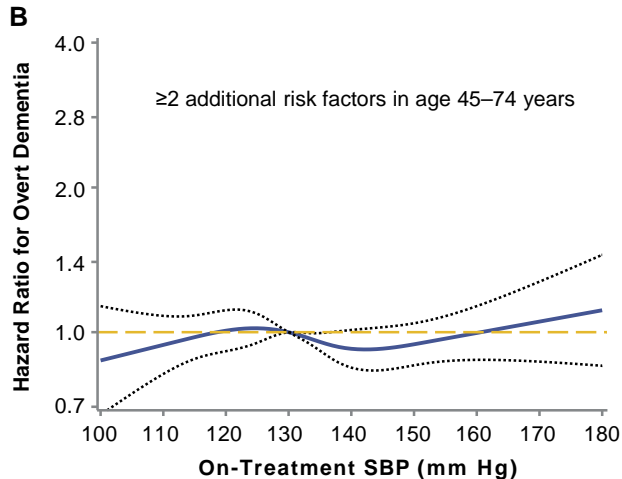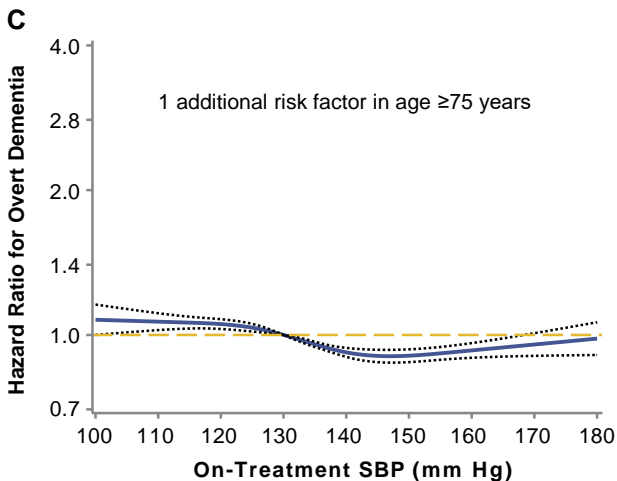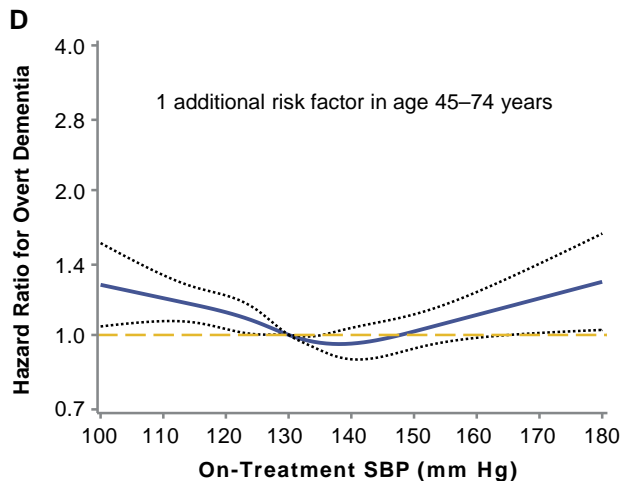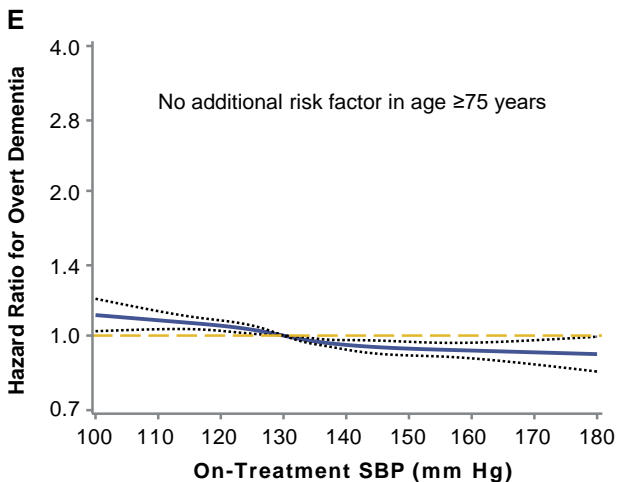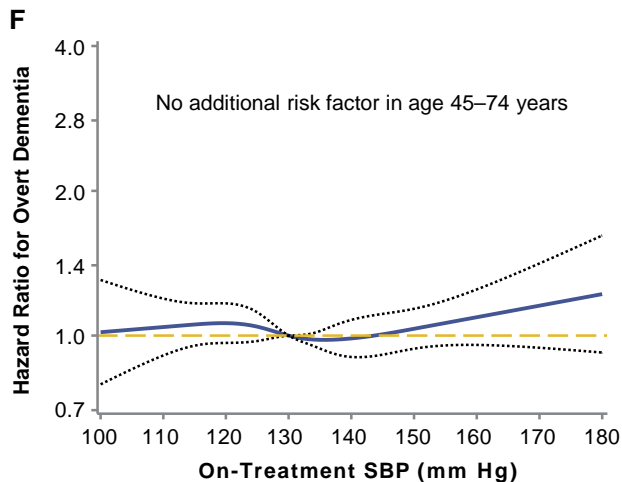

Supplement: S9 Fig — (PDF) [file pone.0297103.s024.pdf]

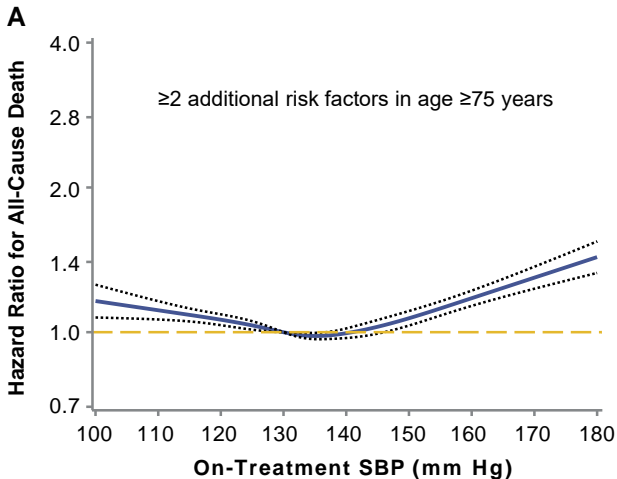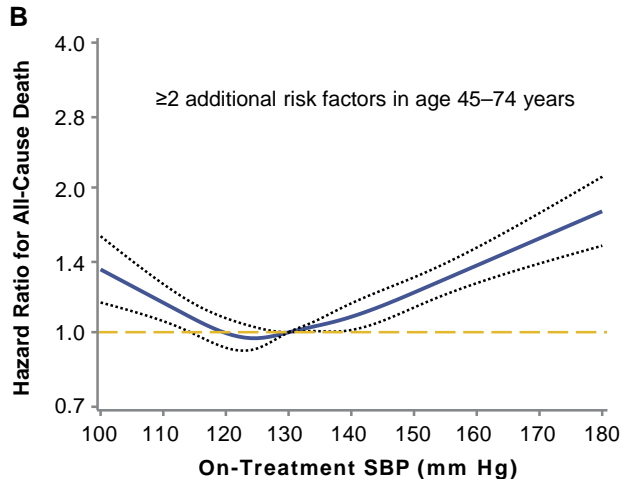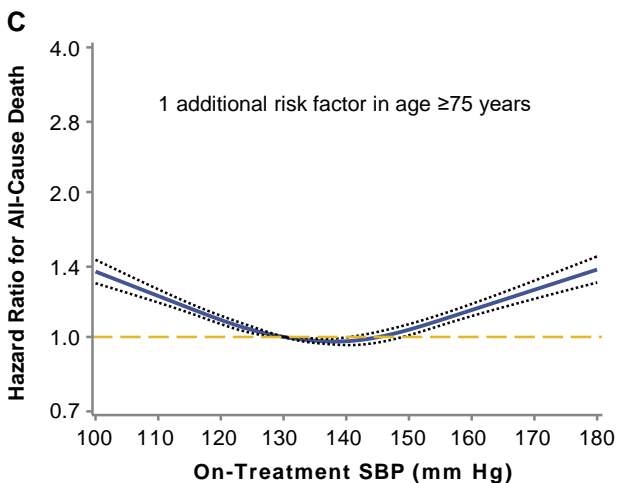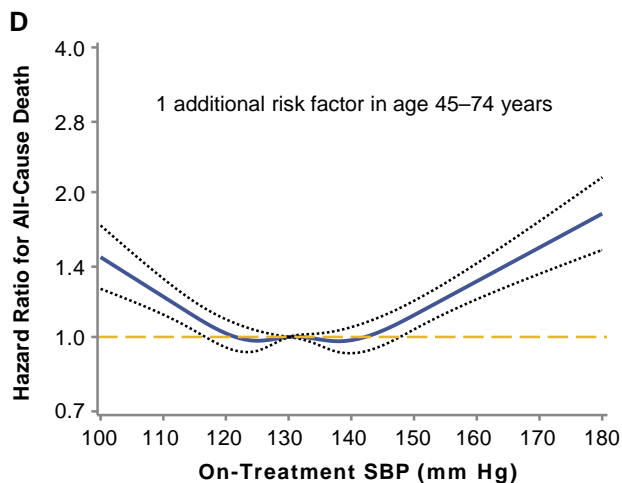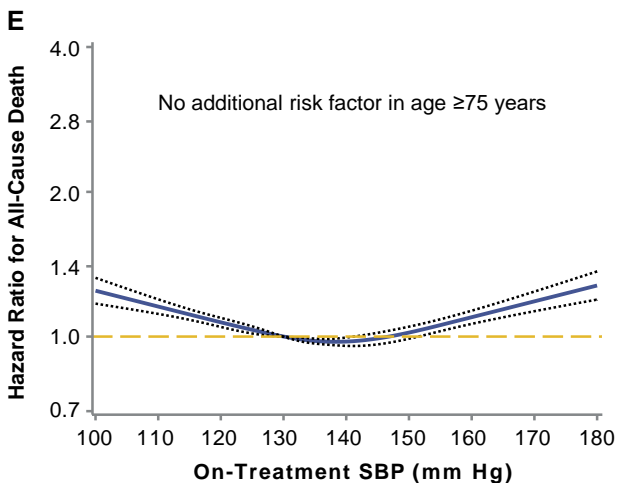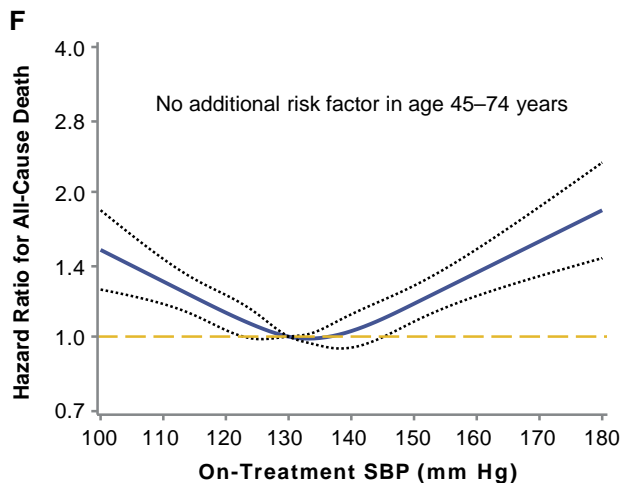

Supplement: S10 Fig — (PDF) [file pone.0297103.s025.pdf]

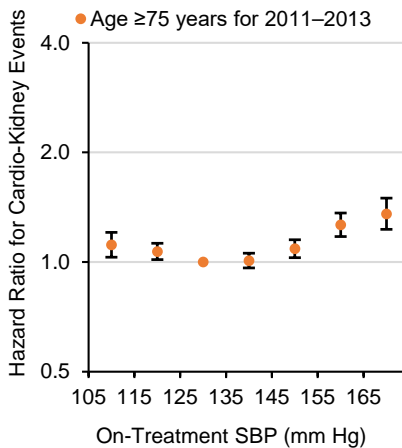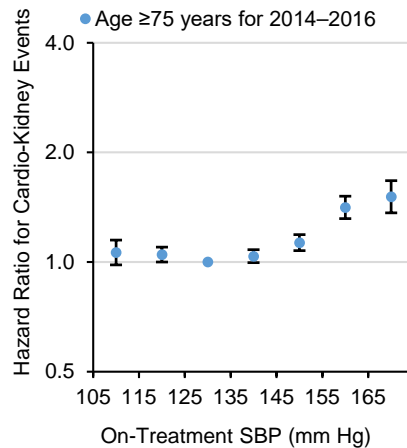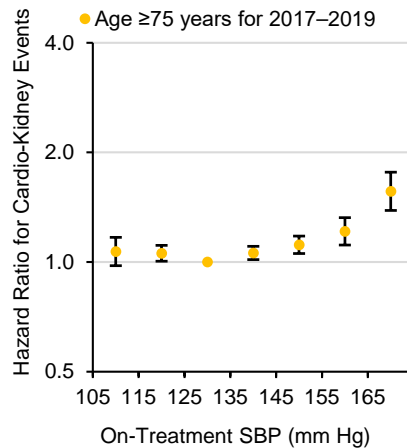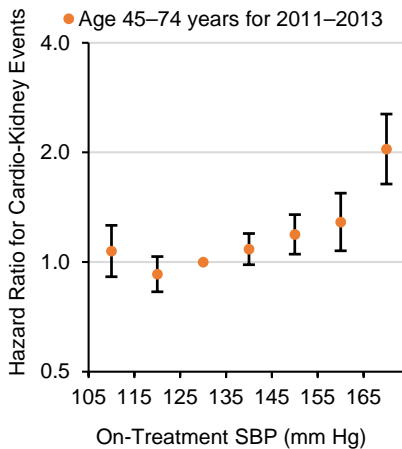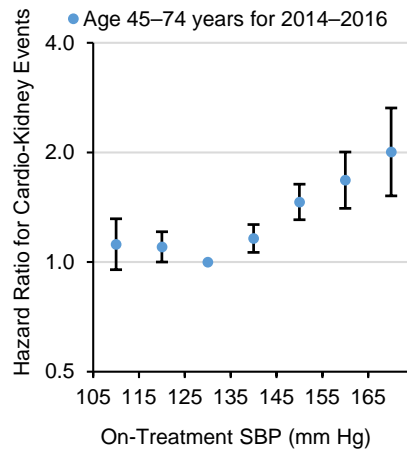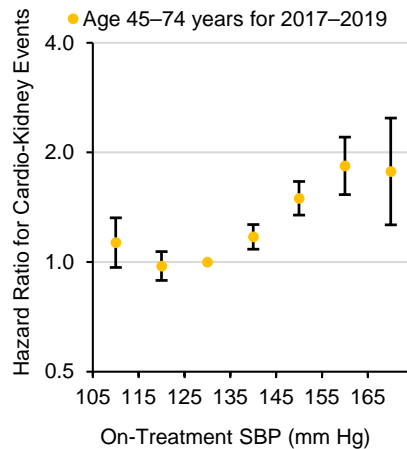

Supplement: S11 Fig — (PDF) [file pone.0297103.s026.pdf]

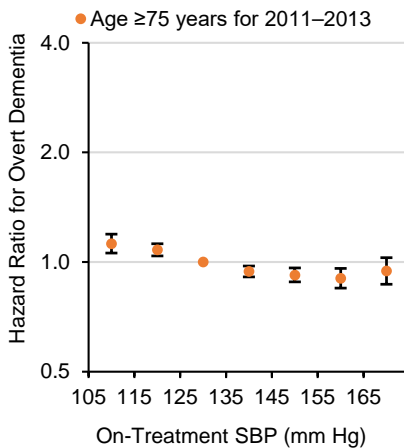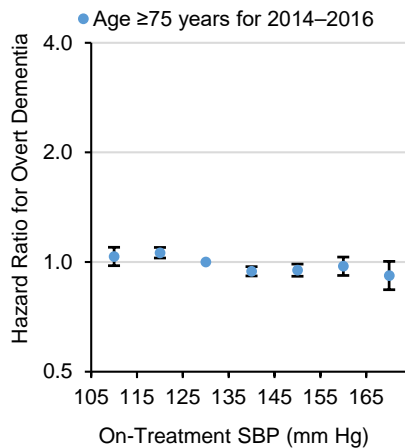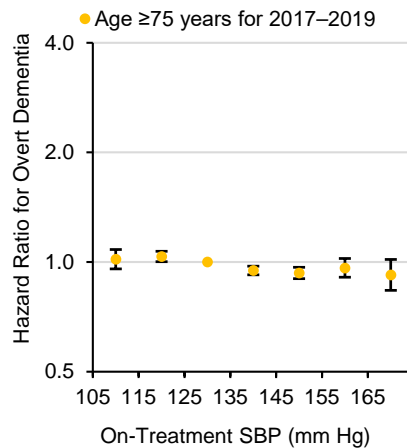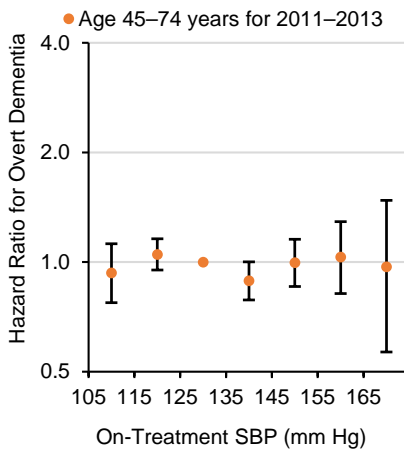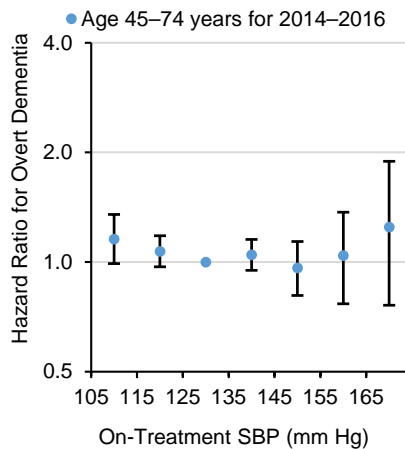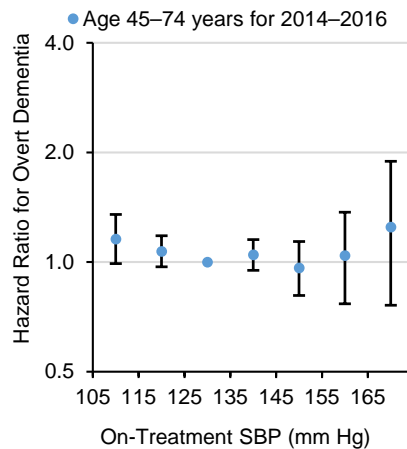

Supplement: S12 Fig — (PDF) [file pone.0297103.s027.pdf]

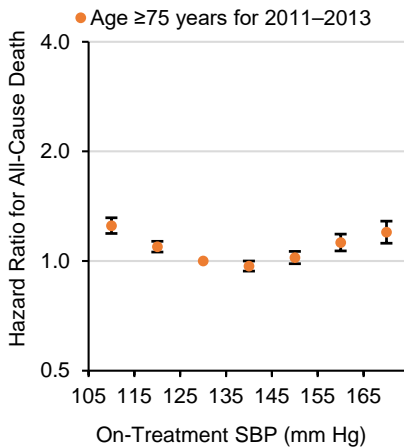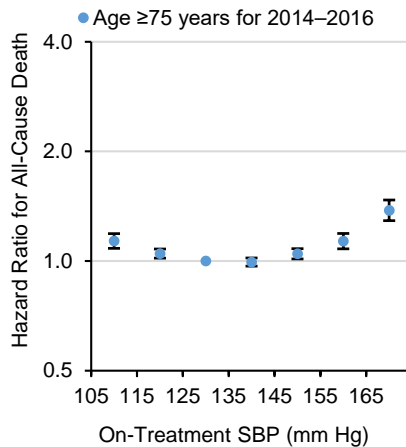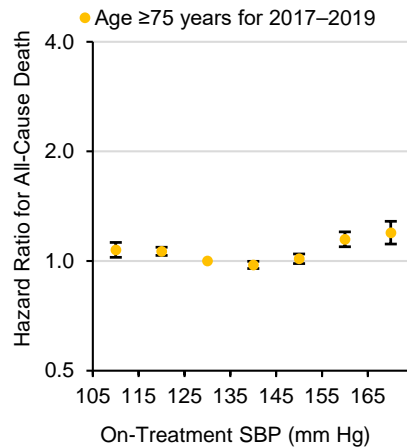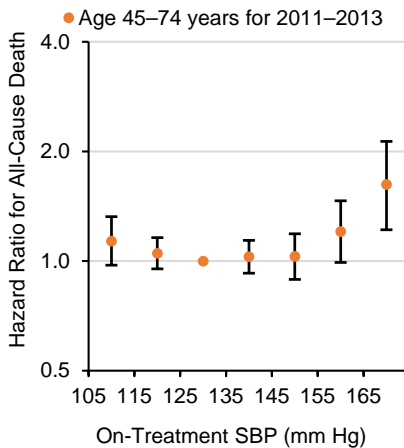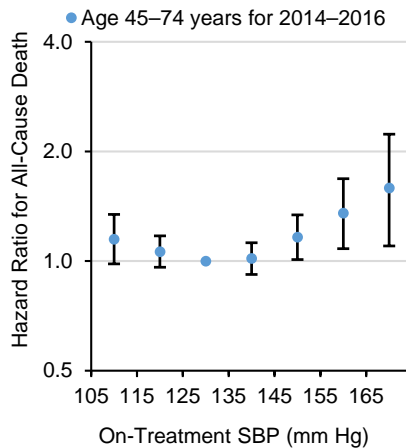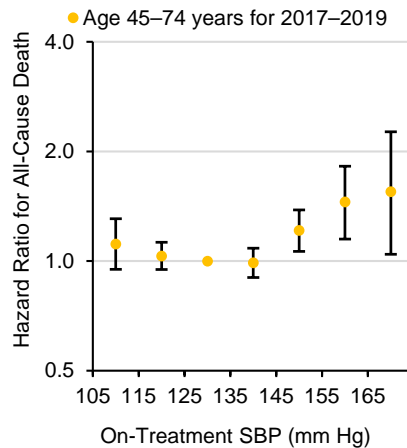

Supplement: S13 Fig — (PDF) [file pone.0297103.s028.pdf]

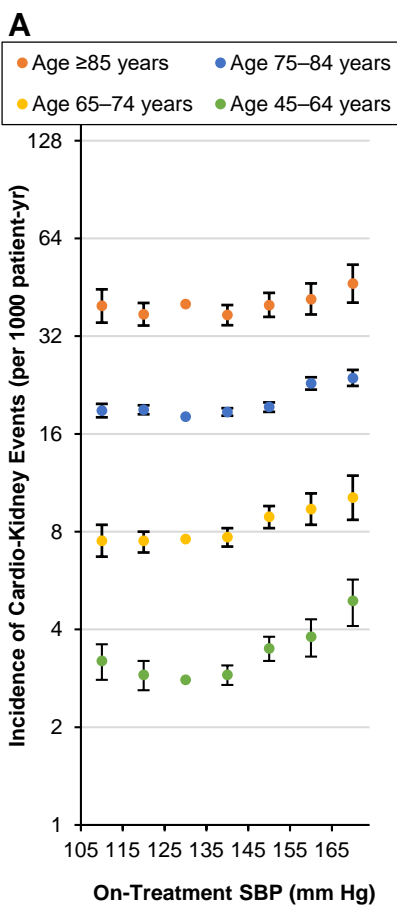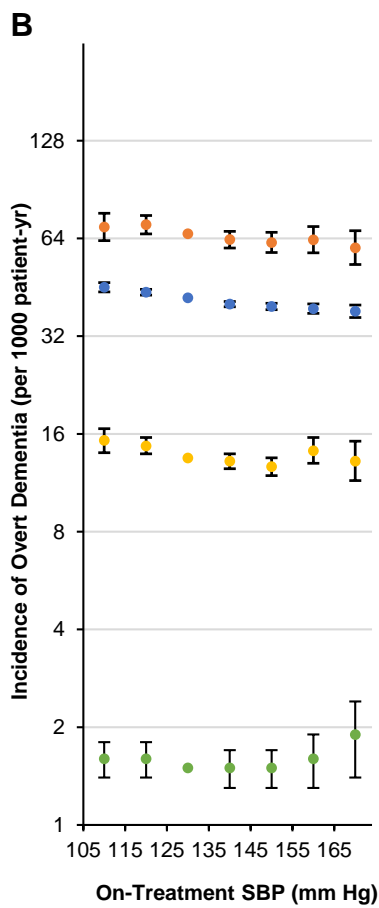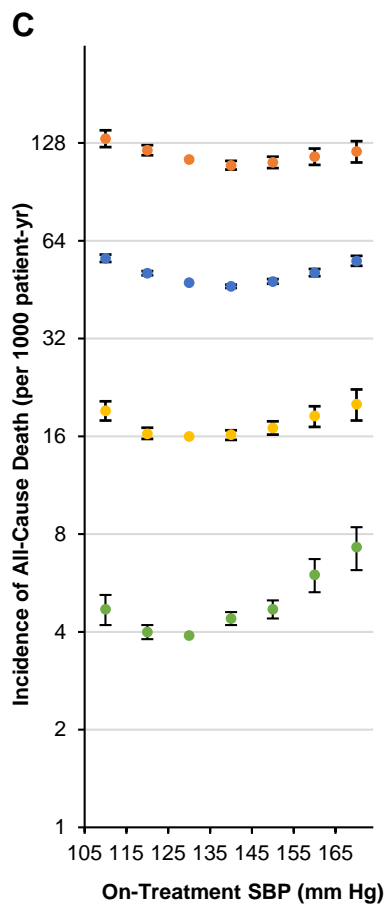

Supplement: S14 Fig — (PDF) [file pone.0297103.s029.pdf]
