# Supplementary material for: Blood pressure control in patients aged above and below 75 years
Source: PLoS One. 2024 Feb 1;19(2):e0297103. doi: 10.1371/journal.pone.0297103 (PMC10833546; doi:10.1371/journal.pone.0297103)
Supplement: S6 Fig — (PDF) [file pone.0297103.s021.pdf]

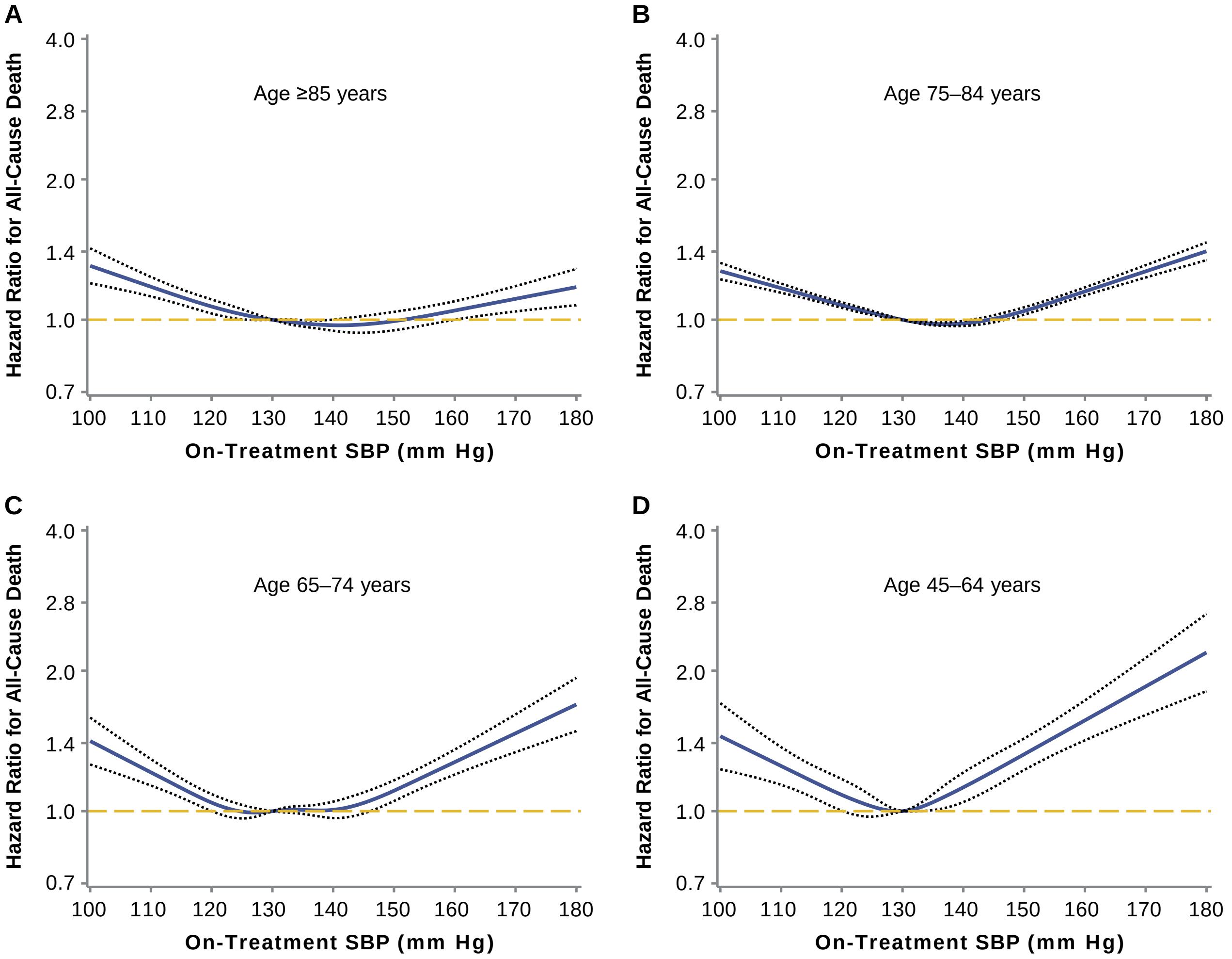
A
Age ≥85 years
B
Age 75–84 years
C
Age 65–74 years
D
Age 45–64 years
Hazard Ratio for All-Cause Death
4.0
2.8
2.0
1.4
1.0
0.7
100
110
120
130
140
150
160
170
180
On-Treatment SBP (mm Hg)
